# Profiling of epidermal lipids in a mouse model of dermatitis: Identification of potential biomarkers

**DOI:** 10.1371/journal.pone.0196595

**Published:** 2018-04-26

**Authors:** Jackeline Franco, Christina Ferreira, Tiago J. Paschoal Sobreira, John P. Sundberg, Harm HogenEsch

**Affiliations:** 1 Department of Comparative Pathobiology, Purdue University, West Lafayette, Indiana, United States of America; 2 Metabolite Profiling Facility, Bindley Bioscience Center, Purdue University, West Lafayette, Indiana, United States of America; 3 The Jackson Laboratory, Bar Harbor, Maine, United States of America; 4 Purdue Institute of Inflammation, Immunology and Infectious Diseases, Purdue University, West Lafayette, Indiana, United States of America; Louisiana State University Health Sciences Center, UNITED STATES

## Abstract

Lipids are important structural and functional components of the skin. Alterations in the lipid composition of the epidermis are associated with inflammation and can affect the barrier function of the skin. SHARPIN-deficient *cpdm* mice develop a chronic dermatitis with similarities to atopic dermatitis in humans. Here, we used a recently-developed approach named multiple reaction monitoring (MRM)-profiling and single ion monitoring to rapidly identify discriminative lipid ions. Shorter fatty acyl residues and increased relative amounts of sphingosine ceramides were observed in *cpdm* epidermis compared to wild type mice. These changes were accompanied by downregulation of the *Fasn* gene which encodes fatty acid synthase. A profile of diverse lipids was generated by fast screening of over 300 transitions (ion pairs). Tentative attribution of the most significant transitions was confirmed by product ion scan (MS/MS), and the MRM-profiling linear intensity response was validated with a C17-ceramide lipid standard. Relative quantification of sphingosine ceramides CerAS(d18:1/24:0)2OH, CerAS(d18:1/16:0)2OH and CerNS(d18:1/16:0) discriminated between the two groups with 100% accuracy, while the free fatty acids cerotic acid, 16-hydroxy palmitic acid, and docosahexaenoic acid (DHA) had 96.4% of accuracy. Validation by liquid chromatography tandem mass spectrometry (LC-MS/MS) of the above-mentioned ceramides was in agreement with MRM-profiling results. Identification and rapid monitoring of these lipids represent a tool to assess therapeutic outcomes in SHARPIN-deficient mice and other mouse models of dermatitis and may have diagnostic utility in atopic dermatitis.

## Introduction

Lipids play an important role in maintaining the integrity of the skin and in inflammatory skin diseases, phototoxicity, and wound healing [1]. They form a critical structural component of the epidermal barrier which prevents water loss and limits the penetration of pathogens, ultraviolet light, and chemicals. The barrier is formed by the outermost layer of the epidermis and consists of anucleated flattened keratinocytes (corneocytes) with abundant keratin filaments cross-linked by envelope proteins embedded in a lamellar lipid matrix [2]. The lipid matrix consists of lipids secreted by terminally differentiated keratinocytes in the granular layer through exocytosis of lamellar bodies along with enzymes that can alter the lipid structure. The main classes of lipids that make up the epidermal lipid matrix are fatty acids, cholesterol esters, and ceramides [2]. Lipids also have antimicrobial activity and can enhance the effect of antimicrobial peptides [3]. Furthermore, lipid mediators play an important role in activation and signaling of innate and adaptive immune cells [1]. Although there is increasing appreciation of the role of lipids in the biology of the skin, knowledge of the lipid composition in healthy and diseased conditions is incomplete.

Atopic dermatitis (AD) is an inflammatory skin disease that affects up to 20% of Caucasian children and 2–10% of adults [4], and greatly impacts the quality of life of patients and their families [5,6]. Atopic dermatitis is a complex disease with a broad spectrum of clinical phenotypes. It has a large heritable component and more than 30 susceptibility genetic loci have been identified [7]. Impairment of the barrier function of the skin and deviation of the immune system are thought to be key components of the pathogenesis of AD. Changes in the lipid barrier may underlie susceptibility to AD, and the inflammation associated with AD can induce changes which sustain and further aggravate the disease [8,9]. These lipid changes are mainly attributed to a decrease of ultra-long chain ceramides and free fatty acids (>26 carbons) with subsequent less dense and less organized lipid lamellae [10]. This creates gaps in the lipid arrangement of the extracellular spaces between the corneocytes [2,11]. However, the exact nature of the changes in the lipid matrix across the spectrum of AD remains to be determined.

Liquid chromatography tandem mass spectrometry (LC-MS/MS) techniques have traditionally been used to quantify the lipid composition in skin and other tissues [12], but these approaches are highly demanding in sample preparation and instrument time, and can only screen for a limited number of lipid features. Therefore, new lipidomic approaches [13,14] that provide an overview of lipid profiles in a faster and more efficient manner could lead to better understanding of these lipid changes and may result in new diagnostic biomarkers to classify disease phenotypes that drive therapeutic development and personalized medicine for AD [15,16]. With the goal of enhancing the knowledge of lipids in the skin and to rapidly identify discriminant lipids, we used an MS analytical strategy named multiple reaction monitoring (MRM)-profiling [17] associated with the monitoring of lipids observed by full mass scan MS as well as free fatty acid profiling by flow injection MS. MRM-profiling is a small molecule discovery workflow performed in two phases. Briefly, the first phase consists of discovery experiments based on neutral loss (NL) and precursor ion (Prec) scan experiments to detect lipids and metabolites in the samples by targeting class-specific chemical motifs such as polar heads of phospholipids or sphingoid bases of ceramides. The second phase of the MRM-profiling is the screening of a larger set of samples for the transitions detected in the discovery phase [18–20]. Thus, the screening phase consists of a profile of the transitions found in the discovery phase for each sample.

Data analysis considers relative amounts of the lipids since the skin barrier lipid metabolism is determined by the relative amounts of different lipids rather than their absolute amounts. The interaction of the lipids themselves is important and this interaction is independent of the cellular total protein content or the tissue weight [20]. The MRM-profiling workflow has been benchmarked in the full mass profiling/fingerprinting screening commonly used for small molecules in ambient ionization and MALDI studies [21,22]. For some ion classes such as free fatty acids (FFA), collision-induced fragmentation is not informative, precluding the use of NL and Prec scans. Therefore, we monitored these in the lipid extracts by single ion monitoring (SIM). We also considered ions present in the full mass scan. All MS experiments were performed using flow injection to a QqQ mass spectrometer with electrospray ionization (ESI) as the ion source.

This study was based on lipid extracts from the epidermis of wild type mice and SHARPIN-deficient *cpdm* mice, which have a spontaneous mutation in exon 1 of the *Sharpin* gene that results in loss of the SHARPIN protein [23]. SHARPIN-deficient mice develop a chronic proliferative dermatitis with morphological and molecular similarities to the intrinsic form of AD. Clinical features include pruritus, progressive alopecia, thickening of the skin, and no increase of total serum IgE [23,24]. Diffuse ortho- and focal parakeratosis is observed along with scattered keratinocyte apoptosis. The dermatitis is characterized by accumulation of eosinophils, mast cells, and type 2 macrophages, and increased expression of cytokines including IL5, IL13, IL33, and TSLP [25–27]. Epidermal samples from wild-type (WT) and *cpdm* mice were subjected to selected Prec and NL scans to profile diverse phospholipids (PL), ceramides, and cholesterol esters (CE). Several hundred transitions were detected in the discovery phase using a subset of animals, and these were used in the screening phase for fast screening of all samples. Data generated clearly discriminated WT and *cpdm* phenotypes based on relative amounts of specific epidermal lipids. A set of discriminating lipids was identified and validated by LC-MS/MS, and comprised three sphingosine ceramides, which could discriminate between WT and *cpdm* mice with 100% accuracy. These lipids will be helpful for the development and assessment of novel therapies in this mouse model. They could also be used to establish and validate a panel of biomarkers for AD in domestic animals and humans to perform patient classification, assess disease progression, and response to treatments.

## Materials and methods

### Mice

36 female C57BL/KaLawRij-*Sharpin*^*cpdm*^*/Sharpin*^*cpdm*^ RijSunJ (*cpdm*) mice and control littermates (WT) were obtained from The Jackson Laboratory and housed at 2 to 4 animals per box with food (Envigo) and water ad libitum. Room temperature was maintained at 20 ± 2 °C and relative humidity at 50 ± 15% with a 12/12 hour light/dark cycle. For the biomarker discovery experimental design, two experiments involving two groups of animals were carried out: the first group (analyzed as a testing set) comprised 7 *cpdm* and 8 WT, and the second group (validation set) had 10 *cpdm* and 11 WT mice. Mice were euthanized at 8 to 9 weeks of age by CO_2_ asphyxiation and cervical dislocation. The animal experiments and procedures were conducted in accordance with the Guide for the Care and Use of Laboratory Animals of the National Institutes of Health. The protocol was approved by the Purdue University Animal Care and Use Committee (PACUC protocol 111001019).

### Sample collection

The skin was shaved, a 2x1 cm skin sample collected from each mouse, and the subcutaneous adipose tissue was removed. The epidermis was separated from the dermis by floating the skin samples in a 5 ml petri dish containing 2.5mL of 500 μg/ml Thermolysin (from *Geobacillus stearothermophilus*, Sigma-Aldrich, St. Louis, MO) supplemented with 10 mM 4-(2- hydroxyethyl)-1-piperazineethanesulfonic acid (HEPES), 132 mM NaCl, 2.7 mM KCl, 0.4 mM NaOH.7H_2_O, 1.8 mM CaCl2.2H_2_O, 1.3 mM MgSO_4_ at pH 7.4 for 2 h at 37°C (adapted from [28]). After incubation, the epidermis was peeled off from the dermis with forceps and stored at -80° until lipid extraction. For gene expression analysis, skin samples were collected and stored in RNAlater (Qiagen, Valencia, CA) at − 80 °C until samples from all replicates were collected.

### Lipid extraction

Samples were individually weighed and 10 mg of dry tissue was homogenized in 2mL vials with 1.4mm ceramic (zirconium oxide) beads with 250uL of water using Precellys24 tissue homogenizer (Bertin Technologies, Rockville, MD, USA). The homogenate was transferred and the Precellys tube was rinsed with 200 μL of methanol (MeOH). The total volume of the homogenate was collected and submitted to lipid extraction using Bligh and Dyer method [29]. By this protocol, phase separation was performed using CHCl_3_/MeOH/H_2_O (1:2:0.8) and the combined organic fractions were centrifuged; the bottom phase was transferred and evaporated. Dried lipid extracts were reconstituted in 40 μL of acetonitrile (ACN)/chloroform at 3:1 volume ratio and stored at -20°C. The reconstituted extracts were individually diluted 50X with ACN/methanol/ammonium acetate 300mM at 3:6.65:0.35 volume ratio and used for MS analysis.

### MRM-profiling

#### Discovery

Samples assigned to the testing set were used for the discovery experiments. The volume of 6μL of lipid extract from individual samples was directly delivered through a micro-autosampler (G1377A) into a QQQ6460 triple quadrupole mass spectrometer (Agilent Technologies, San Jose, CA) equipped with Jet Stream ESI ion source for each of the NL and Prec scans to profile phospholipids [30,31], acylcarnitines [32], cholesterol esters [33,34], ceramides [12,35], diverse fatty acid acyl residues [36], and free fatty acids in positive and negative ion modes (S1 Table). Briefly, phosphatidylcholines were profiled by precursor ion mode of mass-to-charge ratio (*m/z*) 184, and phosphatidylserine (PS), phosphatidylinositol (PI), and phosphatidylethanolamine (PE) were profiled using neutral loss of 185 mass units, 277 mass units, and 141 mass units, respectively. Ceramides were scanned using precursor ion of *m/z* 264.3 for sphingosine ceramides, precursor ion of *m/z* 266.4 for sphinganine ceramides, and precursor ion of *m/z* 282.4 for phytosphingosine ceramides. Two fatty acid acyl residues, oleate and arachidonate, were profiled using neutral loss of 299 and 321 mass units, respectively. Scan for precursor ion of *m/z* 303.1 was used as well for arachidonate acyl residues. Acylcarnitines were detected by precursor ion of *m/z* 85 and cholesterol esters by precursor ion of *m/z* 369.1 and MRM. Cholesterol esters were selected to be monitored instead of free cholesterol because they have a constant ion loss that can be used by MRM profiling for analysis in the same fashion as the other lipids monitored in this study. In contrast, free cholesterol needs derivatization and LC-MS analysis since it does not ionize well and is not associated with either a precursor ion or ion loss that can be monitored by MRM profiling. For FFA profiling, the *m/z* of each free fatty acid was monitored in Q1 and Q3 at the negative ion mode to detect deprotonated FFAs. Values of ion intensity of each lipid ion were normalized by the total ion intensity of each sample. The solvent pumped between injections was ACN + 0.1% formic acid. Initial data processing of the profiles obtained was carried out by using MassHunter (B.06.00).

#### Screening

The 300 molecular features detected in all scans were organized into two methods for targeted lipidomics by flow injection using multiple reaction monitoring (MRM), were each ion was detected by a specific parent and a fragment ion in positive or negative mode. The use of two methods was necessary because of the time and signal requirements to examine all MRMs in a single sample injection. For the MRM scan, the selection for the *m/z* of the parent ion occurs at the first quadrupole (Q1) of a triple quadrupole mass spectrometer, the second quadrupole (Q2) is set to apply collision induced dissociation to cause fragmentation of the parent ion and the third quadrupole (Q3) is set to monitor the fragment. A total of 217 transitions were monitored in the positive and 83 in the negative ion mode (S2 and S3 Tables). These methods were applied to all samples (testing and validation sets, n = 36) so that each sample was individually screened in a high-throughput manner (circa 5 min/sample) by injecting 12μL of lipid extract from each sample into the ESI-MS for the positive ion mode method and 8 μL for the negative ion mode. A blank sample was run in between the samples to avoid carryover. The binary pump flow rate was set at 0.05mL/min, the capillary voltage and the multiplier voltage at the source was 3500 V and 300 V, respectively. For the negative ion mode method, the collision energy voltage was 2 V. Collision energy for the ions detected in positive ion mode varied according to the lipid class as follow: ceramides, PE, lipids with arachidonate acyl residue and oleate acyl residue were set at 22 V, PC and SM at 20 V, PS and PI at 16 V, CE at 17V and acylcarnitines at 30 V. The fragmentation voltage was 100 V for both methods. The raw mass spectrometry data have been deposited in the public proteomics repository MassIVE (http://massive.ucsd.edu) using the identifier: MSV000080197. The data is accessible at ftp://massive.ucsd.edu/MSV000080197. The informative values of *m/z* were tentatively identified by accurate mass measurement against values in online reference databases, the Lipid Maps database (http://www.lipidmaps.org/) and METLIN (https://metlin.scripps.edu), as well as submitted to product ion scan (MS/MS) for attribution confirmation (S1 Fig). The dynamic range and linear ion intensity response of the MRM-profiling were evaluated with C17-ceramide (860517 Avanti Polar Lipids) spiked into 50X diluted pooled epidermis lipid extract. A linear ion intensity response was observed for four orders of magnitude, 1 to 10,000 ppm. Although our experiments were aimed at relative amounts, a calibration curve of C17-ceramide demonstrated excellent linearity and dynamic range exceeding 3 orders of magnitude (S2 Fig).

### LC-MS/MS validation

The validation set of samples (n = 19) were re-extracted following a protocol for high-throughput analysis of sphingolipids by liquid chromatography tandem mass spectrometry (LC-MS/MS) (adapted from [12]). Briefly, samples were homogenized following the above-mentioned procedure with the addition of internal standard of ceramide/sphingolipid mixture I (LM-6002 Advanti Polar Lipids, USA) with 0.5 nmol of each sphingolipid. The total volume of the homogenate was collected and MeOH/CHCl_3_ (2:1) was added. The mixtures were sonicated and incubated overnight at 48 °C in a heating block. After cooling to room temperature, 75μL of 1M KOH in MeOH was added, followed by sonication and incubation for 2 hours at 37 °C in a heating block. The sample was cooled down to room temperature, transferred and evaporated. The extract was reconstituted in 200μL of 80:20 mobile phases RA/RB, where RA is 74:25:1 (v/v/v) of MeOH:H_2_O:FA plus 5nM of ammonium formate and RB is 99:1 (v/v) of MeOH:FA plus 5nM of ammonium formate. The LC column used was 2.1x100 Xbridge C18 (Waters, Milford, MA). The binary pump flow rate was set at 0.3mL/min, the capillary voltage was positive 4000 V and negative 3500 V. The collision energy voltage was 12 V, the fragmentation voltage was 100 and the cell accelerator voltage was 7 V. Seven μL of the reconstituted sample was delivered to the column through a micro-autosampler (G1377A) into a QQQ6460 triple quadrupole mass spectrometer (Agilent Technologies, San Jose, CA) equipped with Jet Stream ESI ion source. The LC column was pre-equilibrated with 100% RA for 1 min. The binary pump was set in a linear gradient to 100% RB in 9 min and held for 3 min. It was then returned to 100% RA in 2 min and re-equilibrated for 5 min. The MRMs (parent-fragment) for the acquisition included the ones found as highly discriminatory by ROC curve analysis (666.3–264.1; 554.3–264.1 and 538.3–264.1). Data processing was carried out by using MassHunter (B.06.00). Concentrations in nmol/mg of tissue were obtained by normalizing by the dried weight of the sample homogenized and by the concentration of the internal standard.

### Quantitative RT-PCR

Quantitative RT-PCR was performed as previously described [37]. RNA was extracted using a Quick-RNA MiniPrep (Zymo Research, Irvine, CA). For each RT-PCR, a 20μl reaction was run with 4μl iScript RT supermix (Bio-Rad Laboratories Inc., Hercules, CA), 100ng RNA template and nuclease free water. For each qPCR, a 10μl reaction was run with 5 μl iTaq Universal Probe SuperMix (2x) (Bio-Rad Laboratories Inc., Hercules, CA), 0.5μl 20x TaqMan Gene Expression Assays primer and probe set for *Gba*, *Pde12*, *Fasn*, and *Elovl1* (ThermoFisher Scientific, Waltham, MA), 1μl cDNA and 3.5μl nuclease-free water. The qRT-PCR was performed at 40 cycles of 95°C for 30 min, 95°C for 15 min and 60 °C for 1 min. Ct values of each gene were normalized by subtracting the Ct values of the housekeeping gene beta-actin (*Actb*) (ΔCt). The relative fold change in mRNA expression between wild-type mice and *cpdm* mice was calculated and expressed as 2^−ΔΔCt^ [38].

### Statistical analysis

The files generated by the mass spectrometer were converted to mzML format using MSConvert (http://proteowizard.sourceforge.net), and an in-house script was developed to obtain the ion intensity of each *m/z* values monitored. Relative amounts of ion abundances were used for statistics. Values of ion intensities for each of the MRMs monitored were normalized by total ion intensity of all MRMs in the method for a given sample. The differences in the mean values for relative amounts of ceramides and free fatty acids were determined by unpaired t-tests with Holm-Sidak correction for multiple comparisons and alpha set at 5% (Graphpad Prism 6.0). Further statistical analysis was performed using MetaboAnalyst 3.0 software (http://www.metaboanalyst.ca) [39]. Data was auto scaled for PCA, volcano plots and heatmaps. The performance of the identified metabolites and their ratios in discriminating WT from *cpdm* samples was evaluated by constructing receiver operating characteristic (ROCs) curves using the testing set and including the validation set as unknowns for classification. Fold change of mRNA expression on the analyzed genes is presented as geometric means with standard error bars. The statistical significance of fold change in *cpdm* to WT mice were calculated by Student’s t-test for unpaired samples.

## Results

### MRM-profiling

Epidermal samples of 7 *cpdm* and 8 wild type (WT) mice were individually subjected to flow injection experiments by ESI-MS in positive and negative ion mode for the discovery of molecular features by chemically supervised scans. Therefore, full mass scans in both polarities, FFA profiling by single ion monitoring (SIM), and Prec and NL scans (S1 Table) targeted to profile phospholipids, cholesterol esters (CE), ceramides (Cer), and acylcarnitines (AC), were used.

Phospholipid profiles were represented by phosphatidylserine (PS), phosphatidylinositol (PI), and phosphatidylethanolamine (PE), which were detected by NL scans of *m/z* 185, *m/z* 277 and *m/z* 141, respectively. Phosphatidylcholine (PC) lipids were profiled by the Prec of *m/z* 184 [30]. Since each phospholipid contains two fatty acids esterified to a glycerol, lipids are attributed by their class abbreviation (PS, PI, PE, PC) followed by the number of carbon atoms in the esterified fatty acid, a colon, and the number of carbon-carbon double bonds in parentheses, such as PC(34:1). The profiles for phospholipids identified 68 molecular features. Cholesterol esters (CE) were screened by the Prec of *m/z* 369.1 [34] and individually screened by the related MRMs as previously described [33], providing a total of 32 molecular features. Acylcarnitines, metabolites essential in fatty acid metabolism, were detected by the Prec of *m/z* 85 [32] yielding 21 transitions. Ceramides were analyzed by three separate Prec scan modes based on the sphingoid base. Sphingosine ceramides (Cer[S]) were detected by the Prec of *m/z* 264.3, dihydroceramide (Cer[DS]) by the Prec of *m/z* 266.4, and phytosphingosine ceramide (Cer[P]) by the Prec of *m/z* 282.4 based on typical fragments as previously reported [12]. A total of 33 molecular features were recovered from these scans. Sixty-three transitions were produced by the NL of *m/z* 299 for oleic acyl residues and the NL of *m/z* 321 and Prec scans of *m/z* 303.1 for arachidonate acyl residues. In the negative ion mode, 83 molecular features were discovered after analysis of FFA by SIM.

The transitions isolated from the discovery methods (S1 Table) as well as the full mass scan ions and FFA as SIM were organized into two fast (2 min of data acquisition) MRM methods, one for each ion mode, for individual sample screening. In total, the discovery methods revealed 217 ions in positive ion mode and 83 ions in negative ion mode as shown in S2 and S3 Tables.

**Fig 1:**
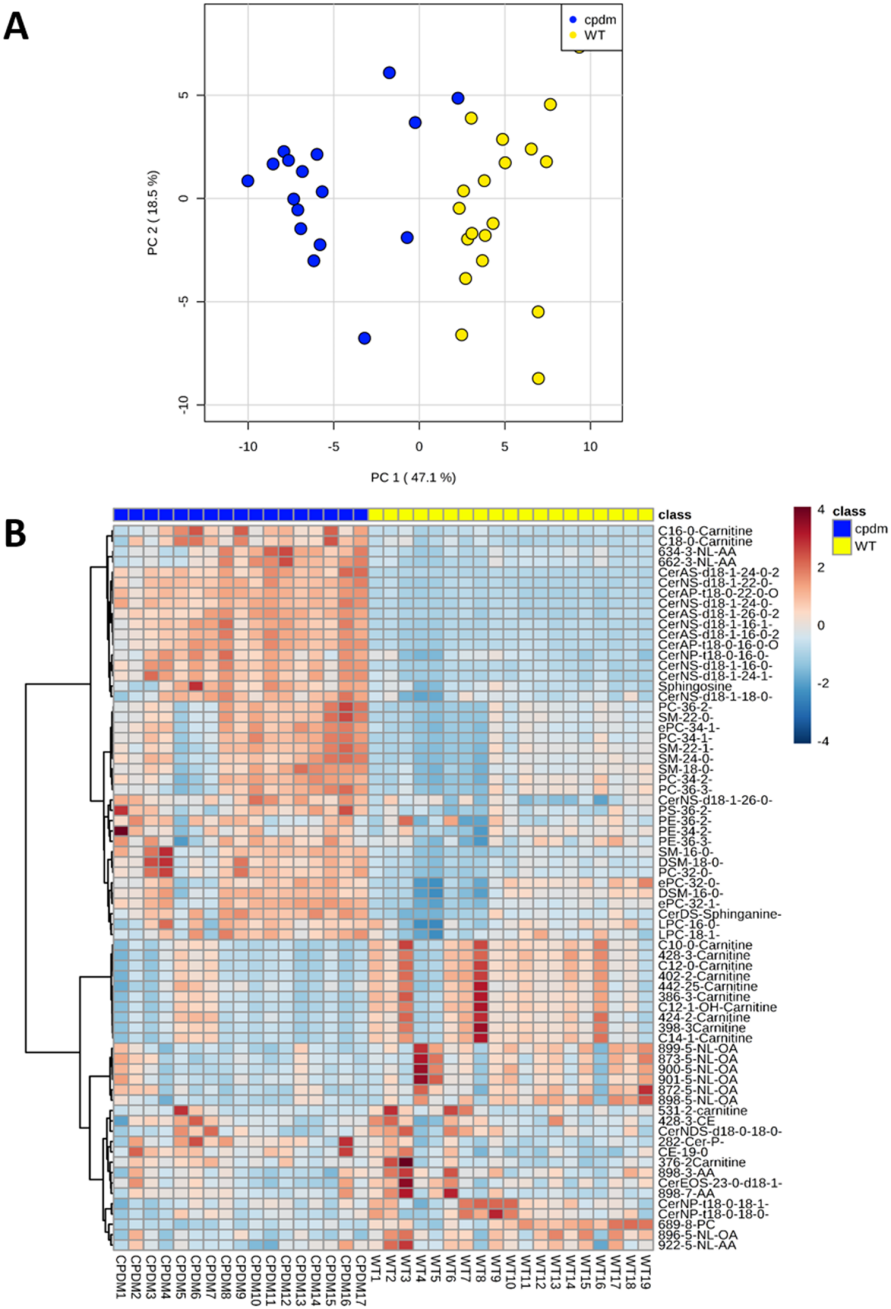
Monitored lipid ions in *cpdm* and WT epidermis by MRM scans in positive ion mode. Clear discrimination of the phenotypes of WT and *cpdm* mouse strains was observed by PCA and cluster analysis. (A) Score plot of principal component analysis (PCA). PC1 explained 47.1% of the variability of the data. When PC2 was included, the explained variance increased to 65.7%. (B) Heat map with the distribution of lipids monitored individually in 36 samples. Lipids not identified are shown with their *m/z* and corresponding lipid class. Color of each cell corresponds to the relative abundance of the lipid feature monitored in the sample.

For the screening phase, a total of 36 samples were used. The lipids were re-extracted from the first group of samples used for the discovery analysis (N = 15; 7 *cpdm* and 8 wild WT) and these samples were considered a testing set (i.e., were used to build a classification system). The new samples (N = 21; 10 *cpdm* and 11 WT) were introduced in the data analysis as a validation set (blind samples). Clear discrimination of the phenotypes of WT and *cpdm* mouse strains was observed by PCA and cluster analysis (Fig 1A). In the positive ion mode, PC1 explained 47.1% of the variability of the data. When PC2 was included, the explained variance increased to 65.7% (S3 Fig). Consistent with the PCA, clustering analysis based on different groups of lipid ions shown as a heat map revealed clear differentiation of *cpdm* from WT mice (Fig 1B).

The structural attribution of the relevant transitions was performed by reference database analysis and by product ion scan (MS/MS) (S1 Fig). Table 1 lists the attribution of significant lipids from the targeted analysis by MRM-profiling as detected by Volcano plot with a *p*-value of 0.05 and at least a two-fold change.

**Table 1:** Tentative attribution of significant molecular features. List of ion pairs (parent and fragment), ion mode of detection, scan description, tentative attribution, fold change (FC) and significance (p-values) resulting from the MRM profiling of WT and *cpdm* mice epidermis. For attribution based on the LipidMaps database, one mass unit has been subtracted of the *m/z* observed in order to obtain the neutral mass of the lipid.

Parent ion	Fragment	Ion Mode	Scan description	Tentative Attribution	Fold Change	p.value
650.3	282.1	[M+H]^+^	Ceramide	CerAP(t18:0/22:0)2OH	5.24	3.67E-21
650.4	264.1	[M+H]^+^	Ceramide	CerNS(d18:1/24:0)	7.31	5.40E-19
622.1	264.1	[M+H]^+^	Ceramide	CerNS(d18:1/22:0)	5.76	4.61E-18
694.15	264.1	[M+H]^+^	Ceramide	CerAS(d18:1/26:0)2OH	4.85	1.54E-17
554.2	264.1	[M+H]^+^	Ceramide	CerAS(d18:1/16:0)2OH	5.23	1.22E-15
554.2	282.1	[M+H]^+^	Ceramide	CerAP(t18:0/16:0)2OH	4.48	6.96E-15
536.1	264.1	[M+H]^+^	Ceramide	CerNS(d18:1/16:1)	4.66	8.34E-15
538.3	264.1	[M+H]^+^	Ceramide	CerNS(d18:1/16:0)	4.89	1.02E-14
666.35	264.1	[M+H]^+^	Ceramide	CerAS(d18:1/24:0)2OH	18.73	1.25E-14
648.4	264.1	[M+H]^+^	Ceramide	CerNS(d18:1/24:1)	3.64	1.64E-12
271.3	271.3	[M-H]^-^	FFA	16-hydroxy(16:0)	2.31	7.67E-12
761.9	184.1	[M+H]^+^	PC	SM(d18:0/20:0) SM(d16:0/22:0) posible isotope PC(34:1)	2.62	4.01E-09
538.2	282.1	[M+H]^+^	Ceramide	CerNP(t18:0/16:0)	2.03	2.21E-08
484	85.1	[M+H]^+^	Acylcarnitine	AC(18:0)	2.73	5.31E-08
703.8	184.1	[M+H]^+^	PC	SM(16:0)	2.41	7.37E-08
456.3	85.1	[M+H]^+^	Acylcarnitine	AC(16:0)	4.02	1.32E-07
438.05	266.1	[M+H]^+^	Ceramide	CerDS(18:0/10:0)	2.26	1.37E-07
746.8	184.1	[M+H]^+^	PC	ePC(34:1) / pPC(34:0)^a^	2.01	7.87E-07
734.8	184.1	[M+H]^+^	PC	PC(32:0)	2.23	9.37E-07
662.3	341.3	[M+H]^+^	NL AA	Not attributted	3.44	1.82E-06
760.8	184.1	[M+H]^+^	PC	PC(34:1)	2.12	2.85E-06
758.8	184.1	[M+H]^+^	PC	PC(34:2)	2.11	5.11E-06
634.3	313.3	[M+H]^+^	NL AA	Not attributed	2.81	6.20E-06
487.5	487.5	[M-H]^-^	FFA	Not attributed	0.42	7.40E-06
689.8	184.1	[M+H]^+^	PC	PG(30:3) SM(d16:1/17:0) SM(d18:1/15:0)	0.50	8.68E-06
788.9	184.1	[M+H]^+^	PC	PC(36:1)	2.02	1.32E-05
787.9	184.1	[M+H]^+^	PC	SM(d18:1/22:0) SM(d16:1/24:0)	2.06	1.95E-05
395.4	395.4	[M-H]^-^	FFA	C26:0	0.23	3.85E-05
372.2	85.1	[M+H]^+^	Acylcarnitine	AC(10:0)	0.49	5.94E-05
400.3	85.1	[M+H]^+^	Acylcarnitine	AC(12:0)	0.48	6.53E-05
414.3	85.1	[M+H]^+^	Acylcarnitine	AC(12:1)OH	0.48	2.63E-04
426.3	85.1	[M+H]^+^	Acylcarnitine	AC(14:1)	0.48	4.44E-04

^a^The ‘e-’prefix is used to indicate the presence of an alkyl ether substituent e.g. ePC(34:1), whereas the ‘p-’prefix is used for the 1Z-alkenyl ether (plasmalogen) substituent e.g. pPC(34:0).

**Fig 2:**
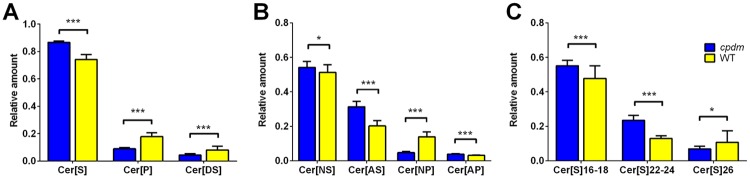
Ceramide profile in *cpdm* and WT epidermis by MRM-profiling. (A) There was an increase of Cer[S] and a decrease of Cer[P] and Cer[DS] in the *cpdm* epidermis. (B) The relative amount of ceramides with α-hydroxy-fatty acid residues was larger in *cpdm* compared to WT. This finding was independent of the sphingoid base as it was observed for both Cer[S] and Cer[P]. (C) Cer[S] carrying fatty acid residues of 16–18 and 22–24 carbons were increased and those with 26 carbons were reduced in *cpdm* samples compared to WT. The vertical axis represents the relative amounts of ceramides detected in the epidermis of *cpdm* and WT mice (horizontal axis). Bars represent the mean +SE of 7 (*cpdm*) or 8 (WT) mice. * p < 0.05; *** p < 0.001, based on unpaired t-test with Holm-Sidak correction for multiple comparisons.

In general, more sphingosine ceramides (Cer[S]) than phytoceramides (Cer[P]) and sphinganine ceramides (Cer[DS]) were detected by MRM-profiling. The overall profile of ceramide composition by sphingoid base showed that relative amounts of phytosphingosine and sphinganine ceramides were decreased in *cpdm* epidermis compared to WT, while sphingosine ceramides were increased. Sphinganine ceramides were omitted from further comparison because of the small amounts in the samples and it was not possible to attribute all detected. The profiling showed a higher proportion of ceramides with hydroxylated fatty acid residues (Cer[AS] or Cer[AP]) in *cpdm* compared to WT. This finding was independent of the sphingoid base, as it was observed for both sphingosines and phytosphingosines. Sphingosine ceramides carrying fatty acid residues of 16–18 and 22–24 carbons were increased in *cpdm* samples compared to WT, while those longer than 26 were reduced (Fig 2).

**Fig 3:**
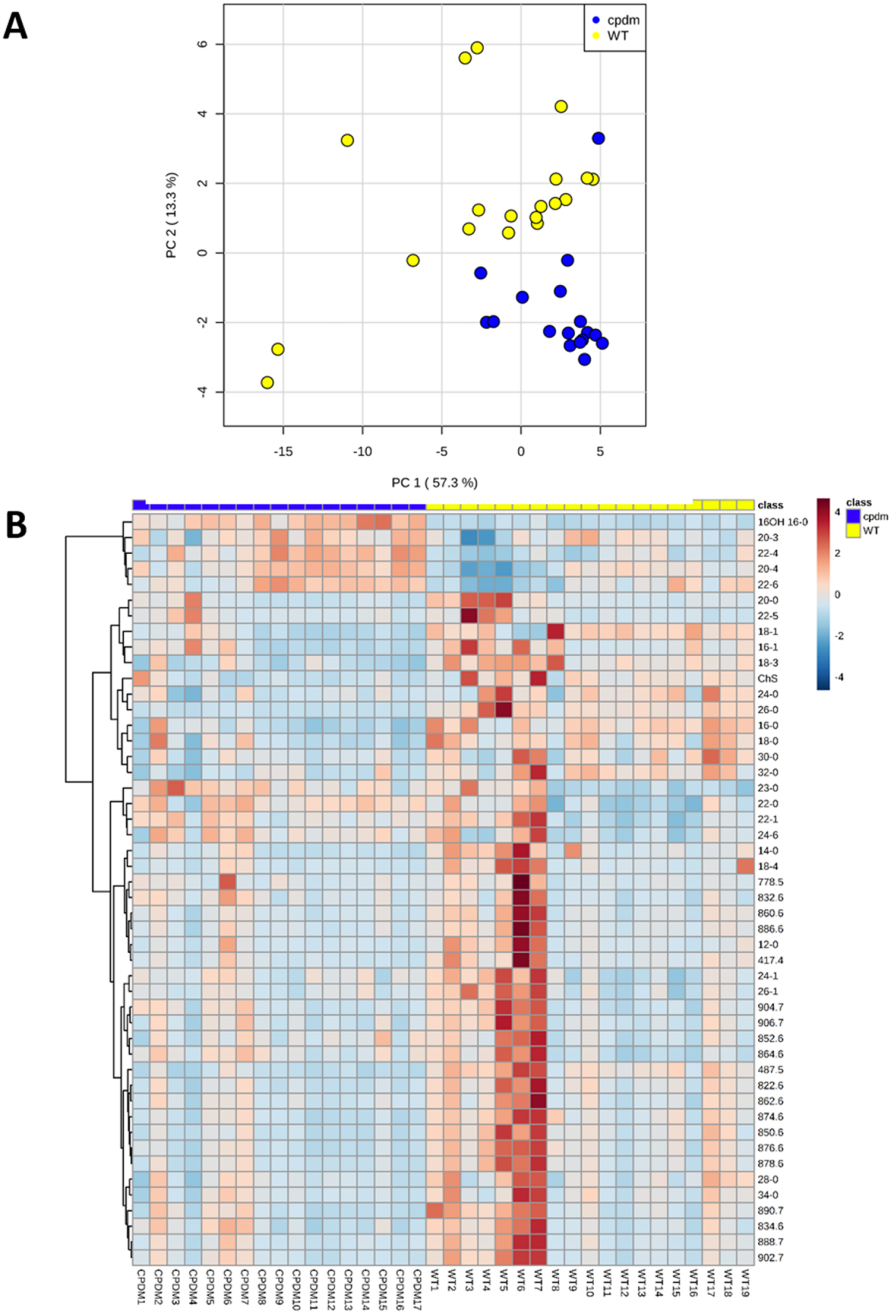
Monitored lipid ions in *cpdm* and WT epidermis by MRM scans in negative ion mode. Clear discrimination of the phenotypes of WT and *cpdm* mouse strains was observed by PCA and cluster analysis. (A) Score plot of principal component analysis (PCA). PC1 explained 57.3% of the variability of the data. When PC2 was included, the explained variance increased to 70.6% (B) Heat map with the distribution of lipids monitored individually in 36 samples. Lipids no identified are shown with their *m/z*. Color of each cell corresponds to the relative abundance of the lipid feature monitored in the samples.

**Fig 4:**
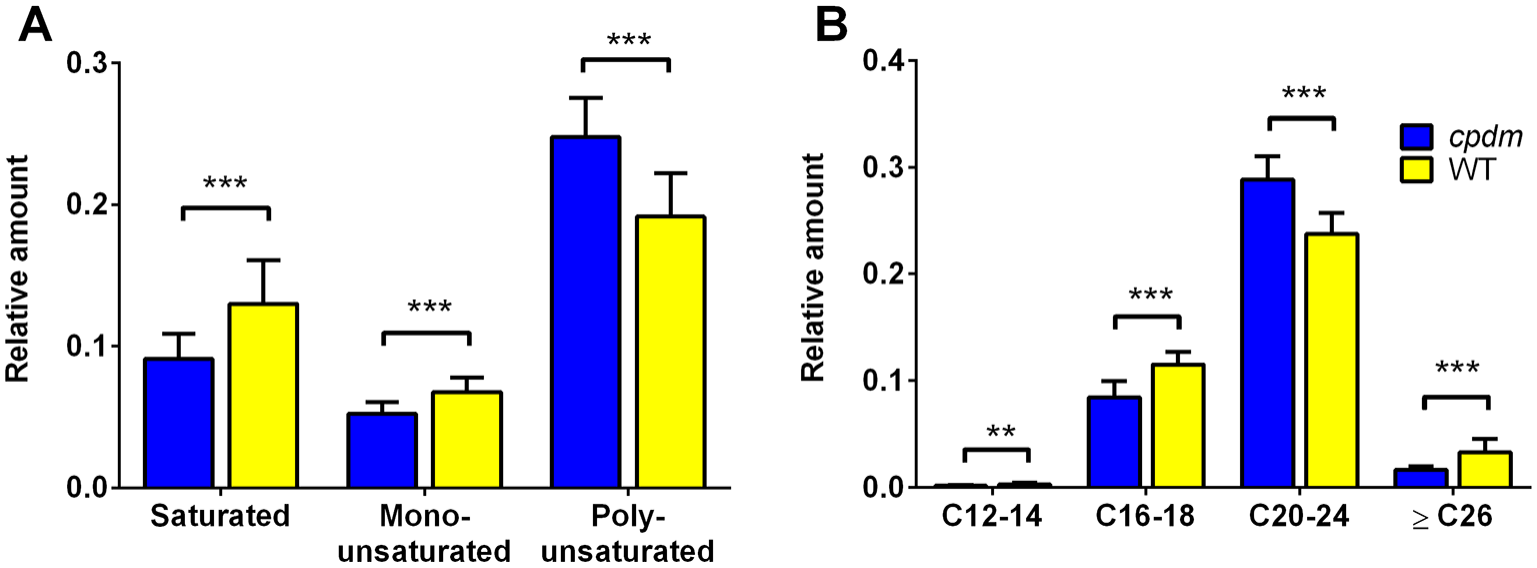
FFA profile in *cpdm* and WT epidermis by MRM-profiling. (A) Relative amounts of polyunsaturated FFAs were increased and saturated and monounsaturated FFAs were decreased in the *cpdm* epidermis. (B) The relative amounts of FFAs with chain length of 12–18 and longer than 26 carbons were reduced in *cpdm* samples compared to WT, instead FFAs with 22–24 carbons were increased. Lipid ions were detected in negative ion mode with *m/z* 199–600 range and normalized by the total ion count. Values are means of 7 (*cpdm)* or 8 (WT) mice. ** p < 0.01; *** p < 0.001, based on unpaired t-test with Holm-Sidak correction for multiple comparisons.

PCA of FFA profiles in negative ion mode revealed an explained variance for PC1 of 57.3% giving a clear separation of the two groups (S4 Fig). The PCA and the heat map suggest that poly-unsaturated fatty acids such as DHA (22:6), AA (20:4), adrenic acid (22:4) and dihomo-γ-linoleic acid (20:3) are determinants of the score plot position of *cpdm* samples and had higher relative ion abundances when compared to WT (Fig 3). In addition, univariate statistics revealed that epidermal samples from WT mice had more saturated and monounsaturated fatty acids while the epidermis of *cpdm* mice contained more polyunsaturated fatty acids. The relative amounts of FFAs with a length of 20–24 carbons were increased in *cpdm* compared to WT, whereas FFAs with 12–18 and longer than 26 carbons were reduced in *cpdm* mice (Fig 4).

### Expression of lipid synthesis enzymes

Based on the results from MRM profiling, we examined the expression levels of two enzymes involved in biosynthesis and elongation of fatty acids, namely fatty acid synthase (FASN) and elongation of very long fatty acids-like 1 (ELOVL1), and two enzymes of the sphingolipid pathway, phosphodiesterase 12 (PDE12) and beta acid glucosidase (GBA). The expression of *Fasn* mRNA was lower in *cpdm* mice compared with WT mice (p < 0.05) (S5 Fig). Changes in the expression of the other enzymes did not reach statistical significance (p > 0.05).

### Receiver operating characteristic (ROC) curve analysis

**Fig 5:**
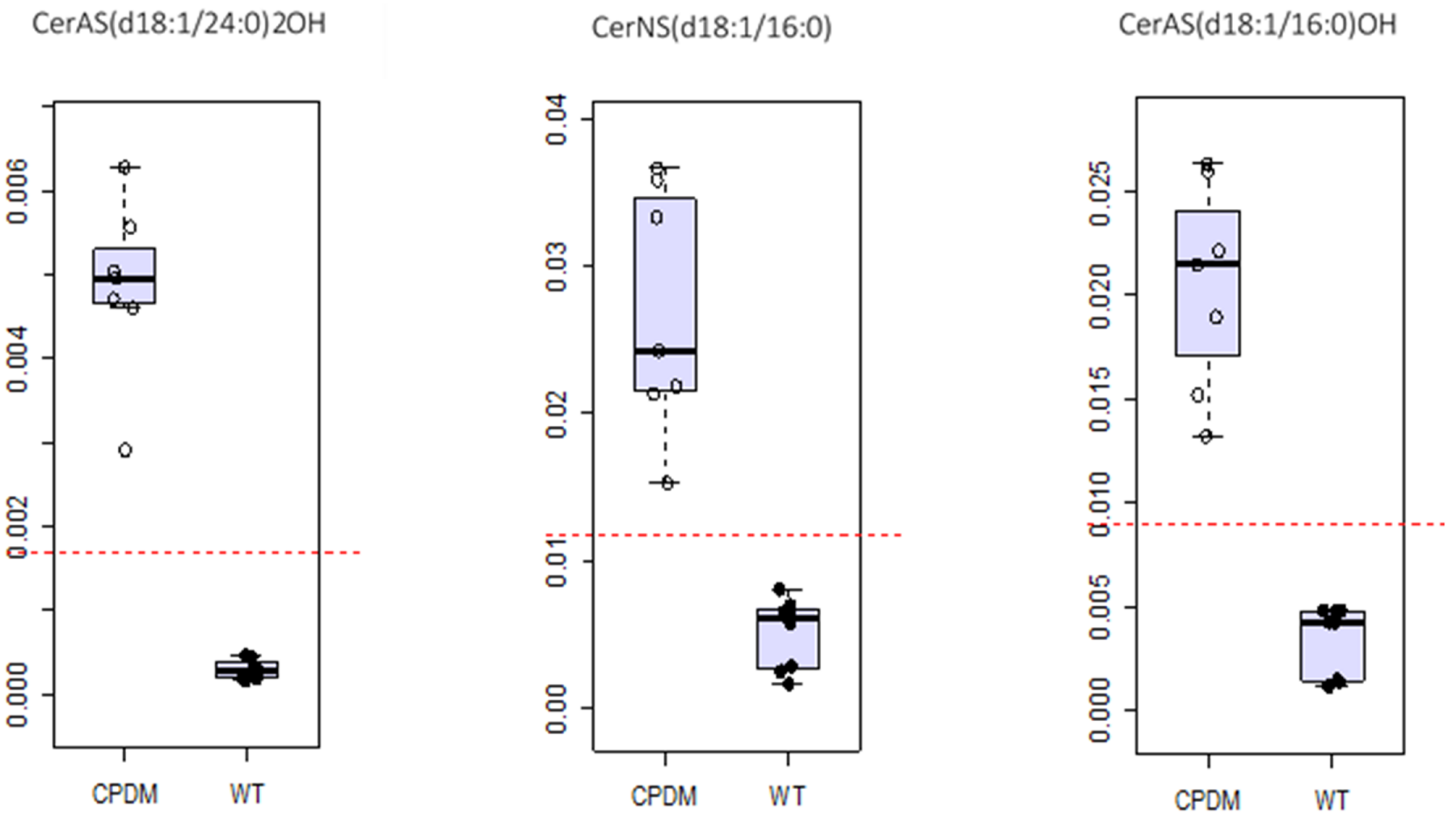
Discriminative value of a set of three ceramides. ROC curve analysis of sphingosine ceramides CerAS(d18:1/24:0)2OH, CerAS(d18:1/16:0)2OH and CerNS(d18:1/16:0) in cpdm and WT epidermis. The threshold (red dotted line) set to differentiate between the two groups is not crossed by any of the samples analyzed for any of the three ceramides.

**Fig 6:**
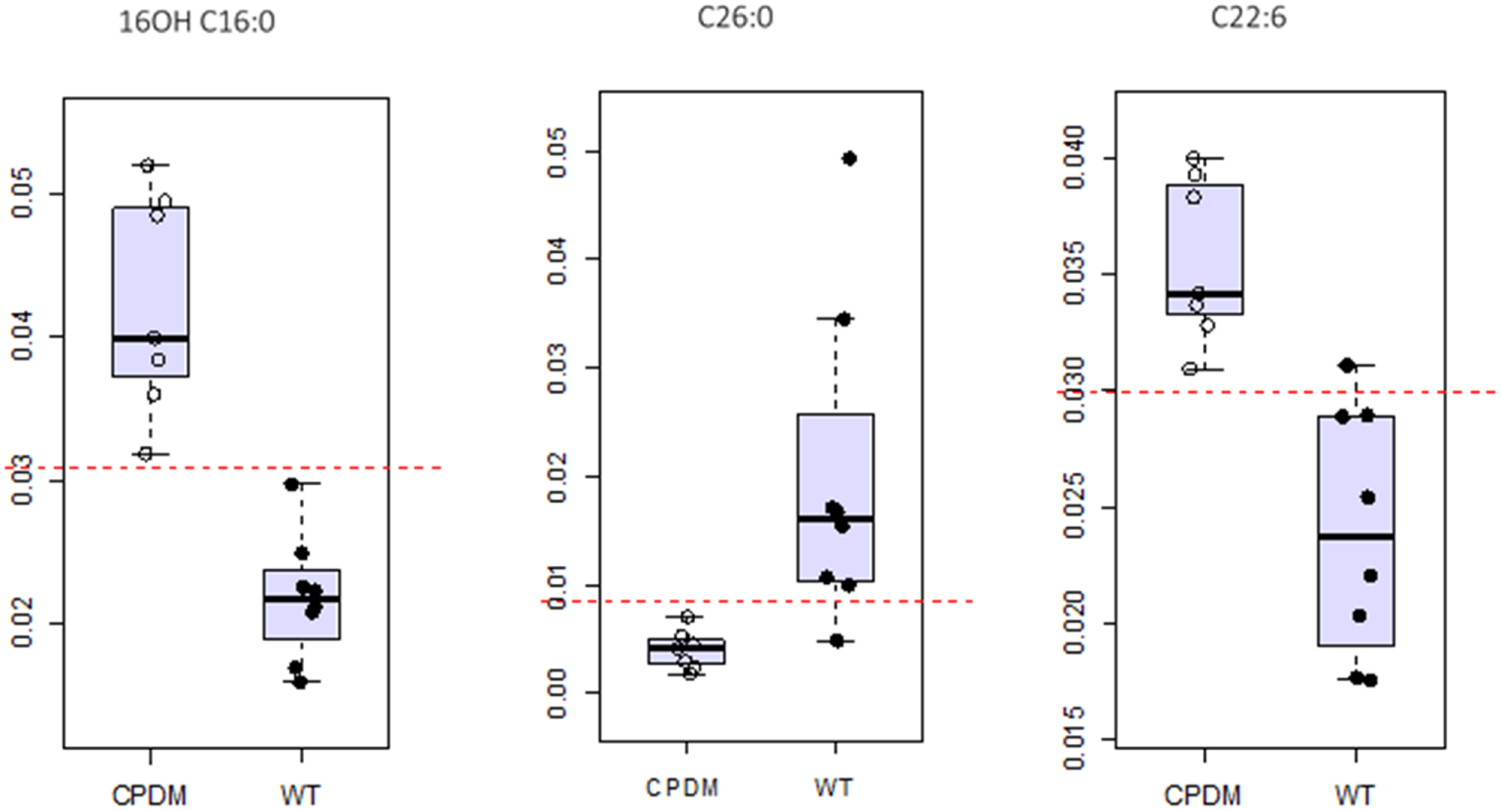
Discriminative value of a set of three free fatty acids. ROC curve analysis of free fatty acids (FFAs) ω-hydroxyl palmitic acid (16OH-16:0), cerotic acid (26:0), and DHA (22:6)in cpdm and WT epidermis. The threshold (red dotted line) set to differentiate between the two groups is crossed by one sample of the WT group analyzed for cerotic acid (26:0), and DHA (22:6). There was no overlap between the groups for ω-hydroxyl palmitic acid (16OH-16:0).

The discriminative values of ceramides and FFA monitored were assessed by developing ROC curves (S4 Table) using the initial test samples (n = 15) to model the classification and the validation set (N = 21) as unknowns. The sphingosine ceramides CerAS(d18:1/24:0)2OH, CerAS(d18:1/16:0)2OH and CerNS(d18:1/16:0) discriminated between WT and *cpdm* mice with 100% accuracy (Fig 5) using partial least square—discriminant analysis (PLS-DA) as the algorithm for the multivariate ROC curve. The area under the curve (AUC) score for the model was 1, and the predicted class probability for the testing samples was precise, with no errors in the attribution (S6 Fig). All new samples were correctly classified with high-predicted probabilities for each sample (>0.99) using random forest (RF) or PLS-DA as algorithms for the multivariate ROC curve (S5 Table). Another ROC was modeled with FFA selected from the targeted negative ion mode method, namely, DHA (22:6), ω-hydroxyl palmitic acid (16OH-16:0) and cerotic acid (26:0). The AUC score for the univariate ROC curve for the training group had a value of 1 for the first two fatty acids and a value of 0.964 for the cerotic acid (Fig 6). The overall model had an AUC of 1 and the class prediction probability of the testing samples was high (S7 Fig). For the new samples there was misclassification of two of the validation set samples using RF and one using PLS-DA, giving an AUC value of 0.964 for the multivariate ROC curve (S6 Table).

The sphingolipids that were selected by ROC curve analysis were analyzed by LC-MS [12] to obtain quantitative results in nmol/mg of tissue. The results were analyzed by ROC curve to confirm the outcome of the MRM-profiling approach. The LC-MS/MS results were in agreement with those obtained by MRM-profiling (S8 Fig).

## Discussion

Changes in the composition and structure of epidermal lipids are found in various skin conditions [1]. In recent years, the epidermal lipid barrier has received most attention in the context of AD [40,41]. Reduced barrier function facilitates penetration by pathogens or irritant molecules that cause an exacerbated inflammatory response characterized by a Th1/Th2 imbalance [42], which in turn can affect the lipid composition and the barrier function of the skin [8,43]. We investigated the lipid composition of the epidermis of *cpdm* mice, a mouse model with histological and immunological characteristics of human inflammatory skin diseases [25,26] applying a straightforward workflow mostly based on MRM-profiling. This exploratory approach focused on relative rather than absolute quantification. This is a widely accepted strategy in discovery MS such as liquid-chromatography-high resolution mass spectrometry [44]. The relative amounts of lipids in the epidermis determine their organization, barrier formation, and biological function [45–47].

The *cpdm* epidermis contained more Cer[S] at the expense of Cer[P] compared with WT mice similar to changes observed in human patients with AD [21,43]. The increase of α-hydroxylated ceramides and reduction of ω-esterified ceramides in *cpdm* mice, similar to changes observed in human AD [48–51], can contribute to reduced membrane stability of keratinocytes [52] and decreased lipid organization and density of the lipid lamellae [53]. Ceramides of 34 carbons were increased in patients with atopic eczema and Netherton syndrome [41,46]. Similarly, an increase of ceramides with 16 carbon fatty acid residues (and 18 carbons from the sphingoid base) was observed in the *cpdm* epidermis. A possible common metabolic pathway of ceramides and FFA was suggested as the fatty acid residues on ceramides are related to the FFA chain lengths, and both FFA and ceramides with chain lengths of 20–24 carbons were present in increased amounts. On the other hand, there was a clear reduction of ceramides and FFA with more than 26 carbons in their acyl chains. This is in agreement with reports of a reduction of ultra-long ceramides and long chain FFA in human AD and another mouse model of AD [8,40,53].

Disruption of the epidermal barrier induces changes in the expression of enzymes required for the biosynthesis of lipids [54]. Conversely, changes in the expression of enzymes may cause changes in the lipid composition of the epidermis. Investigation of the expression of four enzymes involved in the synthesis and of fatty acids and ceramides revealed decreased expression of *Fasn* mRNA, and no significant changes in the other enzymes. Little is known about the effect of inflammation or inflammatory mediators on the expression of lipid synthesis enzymes in the epidermis. Increased immunohistochemical labeling of the lower epidermis for fatty acid synthase was reported in various forms of dermatitis [55]. In addition, treatment of in vitro cultures of human skin with TNF and IL-31 decreased expression of ELOVL1 [8] and cultures with a Th2 cocktail, including IL-4, IL-13 and IL-31 showed significantly lower mRNA expression of ELOVL1, aSmase and GBA [43]. The *cpdm* dermatitis is associated with increased expression of type 2 cytokines, but there was no increase of *Tnf* mRNA in the skin [25].

Sphingomyelin can give rise only to sphingosine ceramides (Cer[AS] and Cer[NS]) [10] in the sphingolipid pathway. Changes in the structure of ceramides and SM have been observed under pathologic conditions including inflammatory diseases [56,57]. Alterations in the length, hydroxylation state and saturation degree of the fatty acid residues can result from inflammation and can also affect the cellular response to inflammatory stimuli [58].

There was an increase of FFA species with 20–24 carbons chain length and a higher degree of unsaturation as reflected in the increase of AA (20:4) and DHA (22:6) and decrease of ultra-long chain fatty acid cerotic acid (C26:0). AA and DHA are important lipid mediators of inflammation having both pro-inflammatory and anti-inflammatory roles [1,59,60]. The FFA profile of the *cpdm* epidermis had fewer fatty acids carrying acyl chains of 12, 14, and 16 carbons. Combined with the significant downregulation of the *Fasn* gene, this indicates alterations in early metabolic pathways in addition to reduced activity of the elongation pathway of the fatty acids. The MRM-profiling in positive mode demonstrated a general increase of PL in the epidermis of *cpdm* mice compared to WT, especially plasmalogens, which can affect the fluidity of cell membranes. Plasmalogens can also incorporate and store AA and DHA which can be released by the action of phospholipase A2 [61,62] suggesting a correlation between the increase of plasmalogens and AA and DHA in the *cpdm* epidermis.

To the best of our knowledge, changes in the structure of acylcarnitines have not been reported in AD. These molecules are involved in fatty acid oxidation disorders, metabolic disease and inflammation [63]. Long chain acylcarnitines (16 and 18 carbons) were increased in *cpdm* epidermis while medium chain (10 and 14 carbons) were reduced. Long chain acylcarnitines can activate NFkB in macrophages resulting in secretion of inflammatory cytokines and chemokines [64]. They may contribute to the dermatitis in *cpdm* mice and may also play a role in atopic dermatitis.

Reports of biomarkers in AD have focused mainly on gene mutations or levels of inflammatory mediators, which vary greatly among individuals and do not allow a clear stratification of patients. For example, filaggrin mutations are only present in a small percentage of AD patients [65,66], disease onset may not depend on it [67] and alteration of lipid processing enzymes are not correlated with presence of *FLG* mutation [9]. Serum biomarkers such as IL31, IL33, and CCL17 had a weak correlation with disease severity [68,69] and do not reliably predict severity as a recent computational model based on 30 serum proteins failed to provide acceptable error values [70]. However, transcriptome analysis in AD patients showed enrichment of pathways related to lipid biosynthesis and metabolism [71] reinforcing the idea that biochemical dysregulation [72] of multiple pathways and gene defects may underlie the pathogenesis of a phenotypically diverse and complex disease such as atopic dermatitis. An unbiased methodology, such as MRM-profiling, is able to capture phenotypic information important for the development of techniques to predict high-risk patients and to discriminate between disease progression stages and treatment response beyond clinical assessment [66,69]. In this study, the prediction model using sphingosine ceramides CerAS(d18:1/24:0)2OH, CerAS(d18:1/16:0)2OH, and CerNS(d18:1/16:0) showed clear discrimination of the samples with a 100% of accuracy. Such information can lead to the identification of biomarkers that will be instrumental in the development of personalized approaches for the treatment of AD [15].

In summary, we report CerAS(d18:1/24:0)2OH, CerAS(d18:1/16:0)2OH, CerNS(d18:1/16:0), cerotic acid, 16-hydroxy palmitic acid, and docosahexaenoic acid (DHA) as highly discriminative lipids in the dermatitis of SHARPIN-deficient mice. The validation set of this panel of biomarkers confirmed its specificity and sensitivity, with an exact class prediction of new samples based on ceramides and a 90.5% success based on FFA. This panel of lipids may be useful as molecular indicators of treatment effect in this and other mouse models of AD. We also suggest that it would be worthwhile to determine whether the amounts of these lipids are altered in the epidermis of human patients and domestic animals with AD.

## Supporting information

S1 Fig Representative added MS/MS spectrum for tentative attribution of transitions selected as potential biomarkers by ROC curve analysis. (A) MS/MS of *m/z* 666.3 corresponding to the sphingosine ceramide Cer(d18:1/24:0)2OH. Three peaks observed correspond to the parent ion (*m/z* 666.3), the release of water with a loss of 18 u (*m/z* 648.3) and the sphingosine base (*m/z* 264.1). The *m/z* difference between *m/z* 648.3 and *m/z* 264.1 correspond to 2-hydroxy-tetracosanoic acid (*m/z* 384.2) (LMFA01050080) (B) MS/MS of *m/z* 538.3 corresponding to the sphingosine ceramide Cer(d18:1/16:0). Three peaks observed correspond to the parent ion (*m/z* 538.3), the release of water with a loss of 18 u (*m/z* 520.2) and the sphingosine base (*m/z* 264.1). The *m/z* difference between *m/z* 520.2 and *m/z* 264.1 correspond to hexadecanoic acid (*m/z* 256.1) (LMFA01010001) (C) MS/MS of *m/z* 554.2 corresponding to the sphingosine ceramide Cer(d18:1/16:0)2OH. Three peaks observed correspond to the parent ion (*m/z* 554.2), the release of water with a loss of 18 u (*m/z* 535.9) and the sphingosine base (*m/z* 264.1). The *m/z* difference between *m/z* 535.9 and *m/z* 264.1 correspond to 2-hydroxy-hexadecanoic acid (*m/z* 271.8) (LMFA01050047). The *m/z* values had a delta +/-0.5. Vertical axis represents the ion intensity response and the horizontal axis is the mass-to-charge (*m/z*) of the ion analized.
(DOCX)

S2 Fig Calibration curve of C17-ceramide lipid standard spiked into pooled lipid extracts from 3 WT and 3 *cpdm* mice. Assay linearity exceeds 3 order of magnitude and has excellent linearity and dynamic range. Five levels were determined in the MassHunter Quantitative Analysis software method for the calibration curve corresponding to concentrations of 1, 10, 100, 1000 and 10000 ppm. 15 points were created out of 3 replicates for each of the 5 levels, all of them were used to plot the curve as shown in the figure. Vertical axis represents the ion intensity response and the horizontal axis is concentration on ppm.
(DOCX)

S3 Fig PCA pair plot of MRM-profiling in positive ion mode. Overview of all combinations for the 5 first principal components (PC) for PCA score plots of MRM profiling data for the method in the positive ion mode (Method 1).
(DOCX)

S4 Fig PCA pair plot of MRM-profiling in negative ion mode. Overview of all combinations for the 5 first principal components (PC) for PCA score plots of MRM profiling data for the method in the negative ion mode (Method 2).
(DOCX)

S5 Fig Expression of enzymes involves in lipid synthesis in the skin. The expression of *Fasn* mRNA was increased significantly increased (* p<0.05) in *cpdm* mice whereas the expression of other enzymes was not changed. The bars represent the mean fold change of mRNA expression in *cpdm* mice versus WT mice (n = 8).
(DOCX)

S6 Fig Discriminative value of a set of three ceramides. (A) ROC curves of sphingosine ceramides CerAS(d18:1/24:0)OH, CerAS(d18:1/16:0)OH and CerNS(d18:1/16:0). (B) Area under the curve (AUC) representation for the testing samples by partial least square—discriminant analysis (PLSA-DA) built with the three selected ceramides; C) Predicted class probability for the testing set of samples of *cpdm* and WT epidermis.
(DOCX)

S7 Fig Discriminative value of a set of three free fatty acids. (A) ROC curves of free fatty acids (FFAs) ω-hydroxyl palmitic acid (16OH-16:0), cerotic acid (26:0), and DHA (22:6); (B) Area under the curve (AUC) representation for the testing samples by partial least square—discriminant analysis (PLSA-DA) built with the three FFAs; (C) Predicted class probability for the testing set of samples of *cpdm* and WT epidermis.
(DOCX)

S8 Fig Discriminative value of a set of three ceramides by LC-MS/MS. (A) ROC curves of sphingosine ceramides CerAS(d18:1/24:0)OH, CerAS(d18:1/16:0)OH and CerNS(d18:1/16:0) in nmol/mg of tissue. The threshold (red dotted line) set to differentiate between the two groups; (B) Area under the curve (AUC) representation for the testing samples by partial least square—discriminant analysis (PLSA-DA) built with the three selected ceramides; C) Predicted class probability for the testing set of samples of *cpdm* and WT epidermis.
(DOCX)

S1 Table Discovery scans. Multidimensional scan modes used for exploratory detection of lipids in *cpdm* and WT epidermis of the testing set.
(DOCX)

S2 Table MRM-profiling method in positive ion mode. List of transitions in the MRM profiling method in positive ion mode (method 1) used to detect the relative amounts of lipids in the samples by the exploratory experiments. Each transition is represented by the *m/z* value of the parent ion, followed by the *m/z* value of the fragment released after collision at Q2.
(DOCX)

S3 Table MRM-profiling method in negative ion mode. List of single ions monitored in the MRM profiling method in negative ion mode (method 2) used to detect the relative amounts of free fatty acids in the lipid extracts from samples.
(DOCX)

S4 Table Biomarker univariate analysis by ROC curve. Tentative attribution of lipids (confirmed by MS/MS experiments), area under the curve (AUC), p-values and log2 fold change (FC) for all ion pairs with AUC scores above 0.5.
(DOCX)

S5 Table Class prediction by ROC curve selected ceramides. Class prediction of the validation set of samples by ROC based on potential ceramide biomarkers.
(DOCX)

S6 Table Class prediction by ROC curve selected FFA. Class prediction of the validation set of samples by ROC based on potential FFA biomarkers.
(DOCX)
