# Supplementary material for: Profiling of epidermal lipids in a mouse model of dermatitis: Identification of potential biomarkers
Source: PLoS One. 2018 Apr 26;13(4):e0196595. doi: 10.1371/journal.pone.0196595 (PMC5919619; doi:10.1371/journal.pone.0196595)
Supplement: S1 Fig — (A) MS/MS of m/z 666.3 corresponding to the sphingosine ceramide Cer(d18:1/24:0)2OH. Three peaks observed correspond to the parent ion (m/z 666.3), the release of water with a loss of 18 u (m/z 648.3) and the sphingosine base (m/z 264.1). The m/z difference between m/z 648.3 and m/z 264.1 correspond to 2-hydroxy-tetracosanoic acid (m/z 384.2) (LMFA01050080) (B) MS/MS of m/z 538.3 corresponding to the sphingosine ceramide Cer(d18:1/16:0). Three peaks observed correspond to the parent ion (m/z 538.3), the release of water with a loss of 18 u (m/z 520.2) and the sphingosine base (m/z 264.1). The m/z difference between m/z 520.2 and m/z 264.1 correspond to hexadecanoic acid (m/z 256.1) (LMFA01010001) (C) MS/MS of m/z 554.2 corresponding to the sphingosine ceramide Cer(d18:1/16:0)2OH. Three peaks observed correspond to the parent ion (m/z 554.2), the release of water with a loss of 18 u (m/z 535.9) and the sphingosine base (m/z 264.1). The m/z difference between m/z 535.9 and m/z 264.1 correspond to 2-hydroxy-hexadecanoic acid (m/z 271.8) (LMFA01050047). The m/z values had a delta +/-0.5. Vertical axis represents the ion intensity response and the horizontal axis is the mass-to-charge (m/z) of the ion analized. (DOCX) [file pone.0196595.s001.docx]

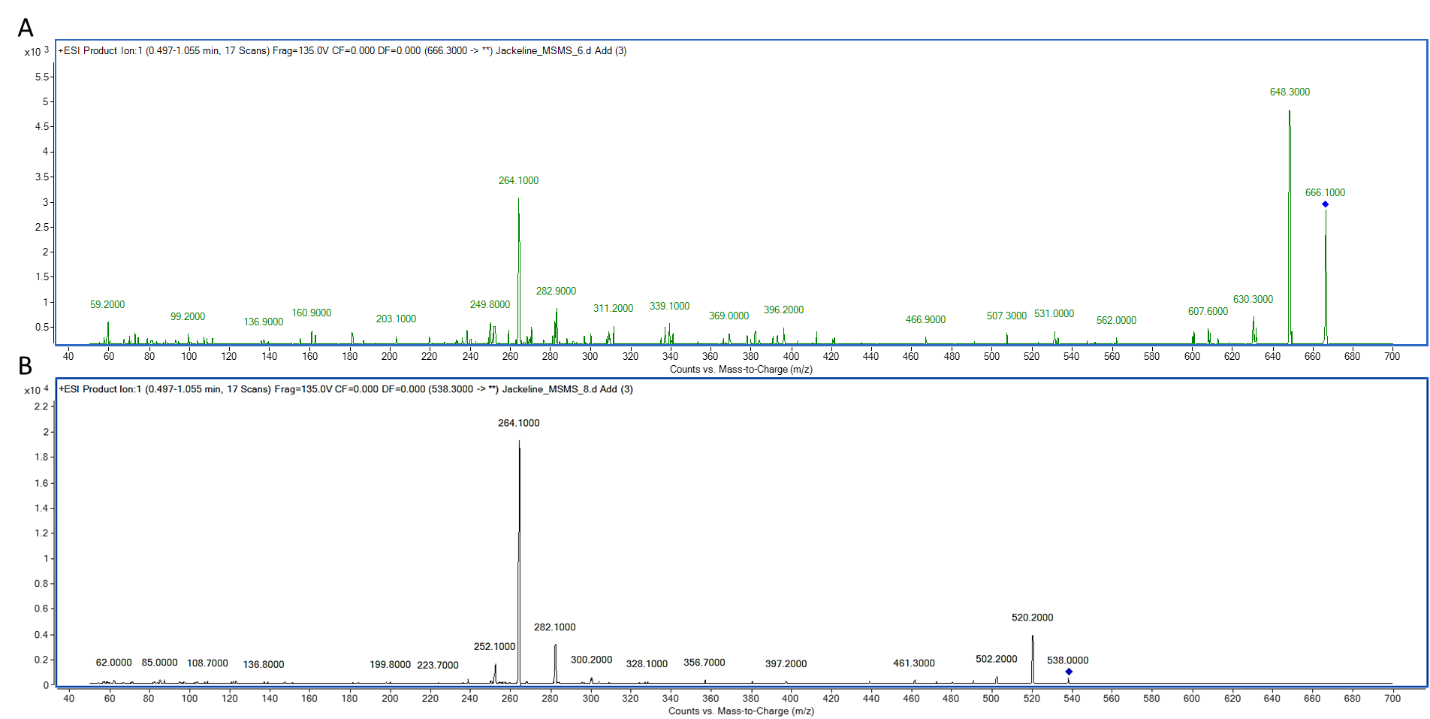

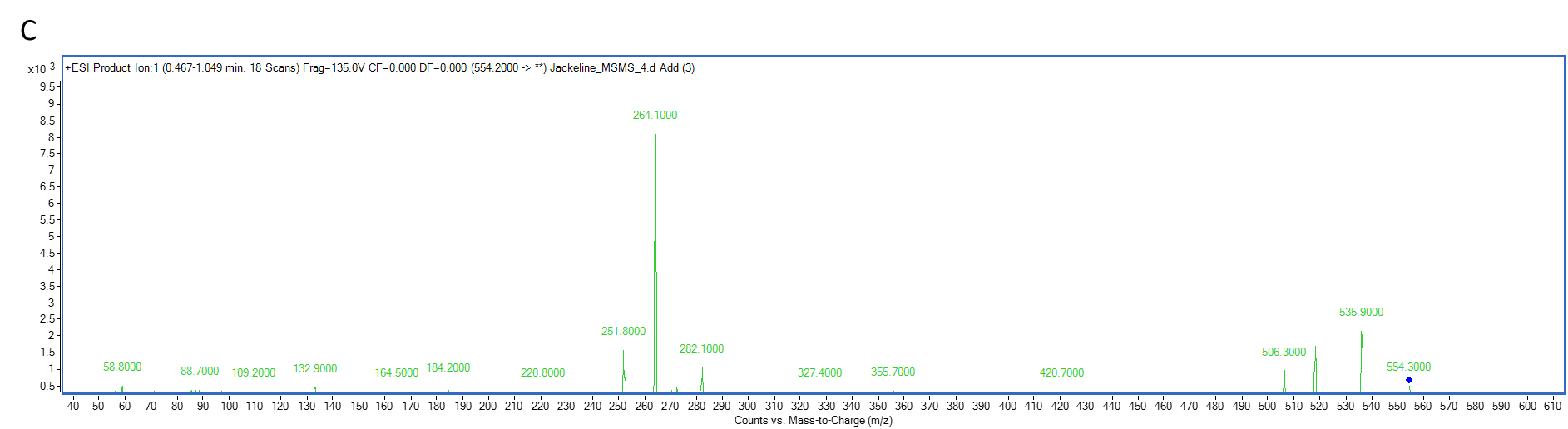


**S1 Fig. Representative MS/MS spectrum for tentative attribution of transitions selected as potential biomarkers by ROC curve analysis.** (A) MS/MS of m/z 666.3 corresponding to the sphingosine ceramide Cer(d18:1/24:0)2OH. Three peaks observed correspond to the parent ion (m/z 666.3), the release of water with a loss of 18 u (m/z 648.3) and the sphingosine base (m/z 264.1). The m/z difference between m/z 648.3 and m/z 264.1 correspond to 2-hydroxy-tetracosanoic acid (m/z 384.2) (LMFA01050080) (B) MS/MS of m/z 538.3 corresponding to the sphingosine ceramide Cer(d18:1/16:0). Three peaks observed correspond to the parent ion (m/z 538.3), the release of water with a loss of 18 u (m/z 520.2) and the sphingosine base (m/z 264.1). The m/z difference between m/z 520.2 and m/z 264.1 correspond to hexadecanoic acid (m/z 256.1) (LMFA01010001) (C) MS/MS of m/z 554.2 corresponding to the sphingosine ceramide Cer(d18:1/16:0)2OH. Three peaks observed correspond to the parent ion (m/z 554.2), the release of water with a loss of 18 u (m/z 535.9) and the sphingosine base (m/z 264.1). The m/z difference between m/z 535.9 and m/z 264.1 correspond to 2-hydroxy-hexadecanoic acid (m/z 271.8) (LMFA01050047). The m/z values had a delta +/-0.5. Vertical axis represents the ion intensity response and the horizontal axis is the mass-to-charge (m/z) of the ion analyzed.
