# Supplementary material for: Profiling of epidermal lipids in a mouse model of dermatitis: Identification of potential biomarkers
Source: PLoS One. 2018 Apr 26;13(4):e0196595. doi: 10.1371/journal.pone.0196595 (PMC5919619; doi:10.1371/journal.pone.0196595)
Supplement: S2 Fig — Assay linearity exceeds 3 order of magnitude and has excellent linearity and dynamic range. Five levels were determined in the MassHunter Quantitative Analysis software method for the calibration curve corresponding to concentrations of 1, 10, 100, 1000 and 10000 ppm. 15 points were created out of 3 replicates for each of the 5 levels, all of them were used to plot the curve as shown in the figure. Vertical axis represents the ion intensity response and the horizontal axis is concentration on ppm. (DOCX) [file pone.0196595.s002.docx]

**
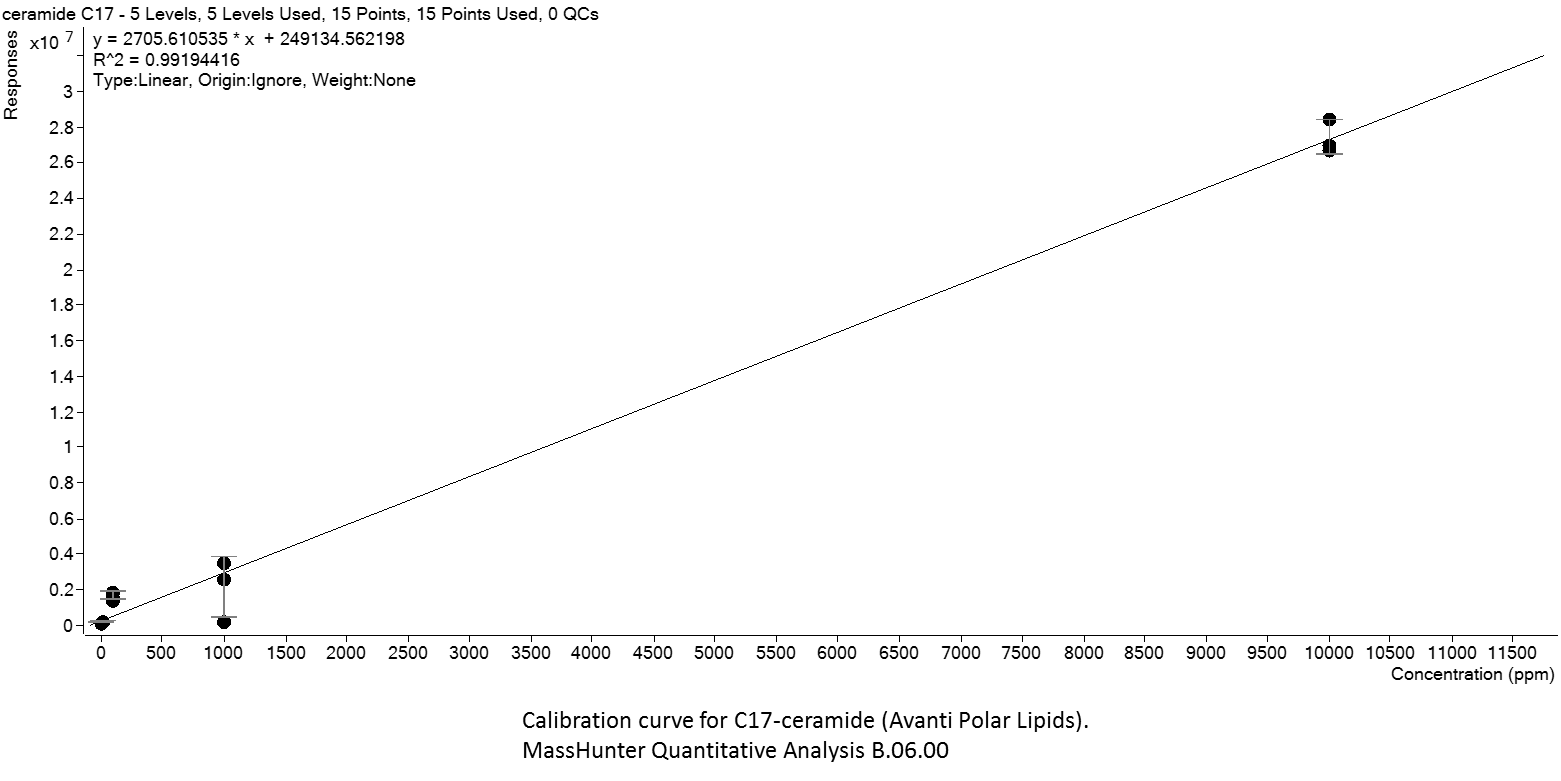
**

**S2 Fig. Calibration curve of C17-ceramide lipid standard spiked into pooled lipid extracts from 3 WT and 3 *cpdm* mice**. Assay linearity exceeds 3 order of magnitude.. Five levels were determined in the MassHunter Quantitative Analysis software method for the calibration curve corresponding to concentrations of 1, 10, 100, 1000 and 10000 ppm. 15 points were created out of 3 replicates for each of the 5 levels, all of them were used to plot the curve as shown in the figure. Vertical axis represents the ion intensity response and numbers on the horizontal axis refer to the concentration (ppm).
