# Supplementary material for: Profiling of epidermal lipids in a mouse model of dermatitis: Identification of potential biomarkers
Source: PLoS One. 2018 Apr 26;13(4):e0196595. doi: 10.1371/journal.pone.0196595 (PMC5919619; doi:10.1371/journal.pone.0196595)
Supplement: S3 Fig — Overview of all combinations for the 5 first principal components (PC) for PCA score plots of MRM profiling data for the method in the positive ion mode (Method 1). (DOCX) [file pone.0196595.s003.docx]

**S3 Fig. PCA pair plot of MRM-profiling in positive ion mode.** Overview of all combinations for the 5 first principal components (PC) for PCA score plots of MRM profiling data for the method in the positive ion mode (Method 1).
