# Supplementary material for: Profiling of epidermal lipids in a mouse model of dermatitis: Identification of potential biomarkers
Source: PLoS One. 2018 Apr 26;13(4):e0196595. doi: 10.1371/journal.pone.0196595 (PMC5919619; doi:10.1371/journal.pone.0196595)
Supplement: S5 Fig — The expression of Fasn mRNA was increased significantly increased (* p<0.05) in cpdm mice whereas the expression of other enzymes was not changed. The bars represent the mean fold change of mRNA expression in cpdm mice versus WT mice (n = 8). (DOCX) [file pone.0196595.s005.docx]

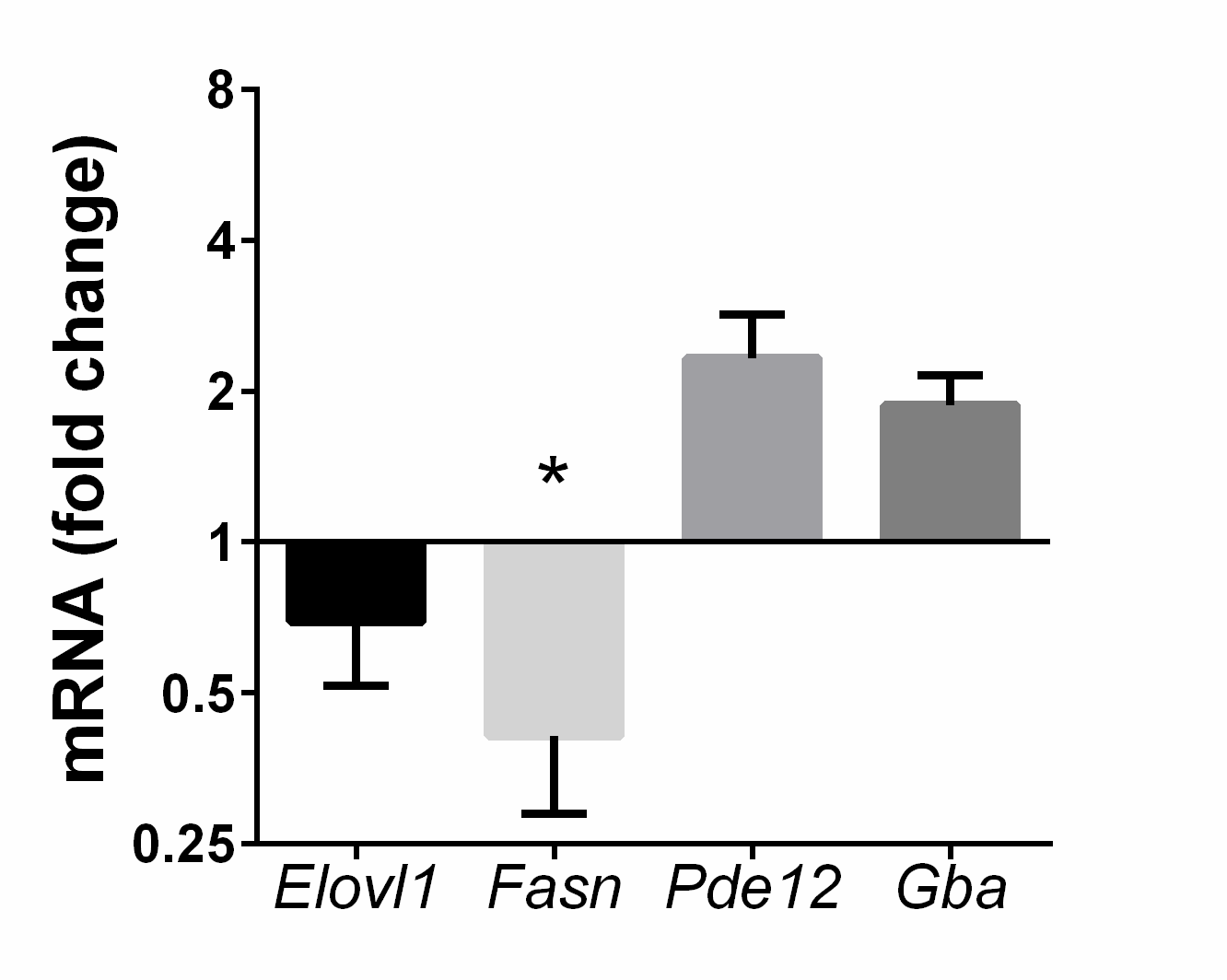


**S5 Fig. Expression of enzymes involves in lipid synthesis in the skin.** The expression of *Fasn* mRNA was increased significantly increased (*p<0.05) in *cpdm* mice whereas the expression of other enzymes was not changed. The bars represent the mean fold change of mRNA expression in *cpdm* mice versus WT mice (n=8).
