# Supplementary material for: Profiling of epidermal lipids in a mouse model of dermatitis: Identification of potential biomarkers
Source: PLoS One. 2018 Apr 26;13(4):e0196595. doi: 10.1371/journal.pone.0196595 (PMC5919619; doi:10.1371/journal.pone.0196595)
Supplement: S6 Fig — (A) ROC curves of sphingosine ceramides CerAS(d18:1/24:0)OH, CerAS(d18:1/16:0)OH and CerNS(d18:1/16:0). (B) Area under the curve (AUC) representation for the testing samples by partial least square—discriminant analysis (PLSA-DA) built with the three selected ceramides; C) Predicted class probability for the testing set of samples of cpdm and WT epidermis. (DOCX) [file pone.0196595.s006.docx]

**
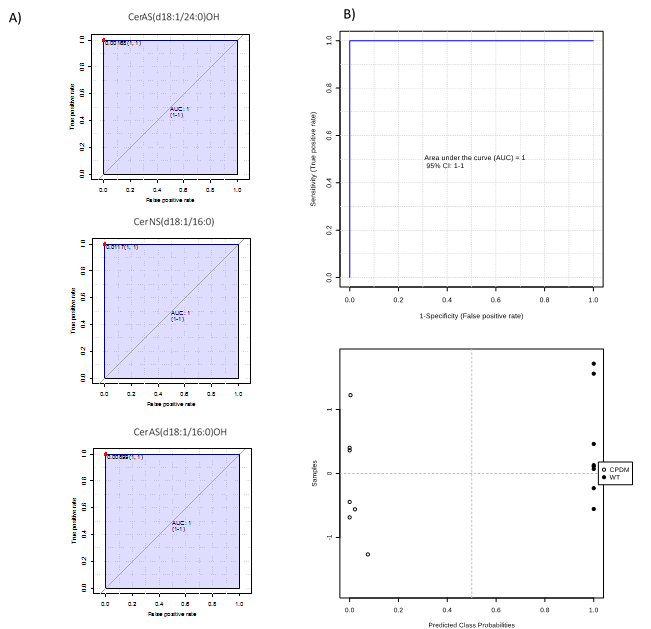
**

**S6 Fig. Discriminative value of a set of three ceramides.** (A) ROC curves of sphingosine ceramides CerAS(d18:1/24:0)OH, CerAS(d18:1/16:0)OH and CerNS(d18:1/16:0). (B) Area under the curve (AUC) representation for the testing samples by partial least square – discriminant analysis (PLSA-DA) built with the three selected ceramides; C) Predicted class probability for the testing set of samples of *cpdm* and WT epidermis.
