# Supplementary material for: Profiling of epidermal lipids in a mouse model of dermatitis: Identification of potential biomarkers
Source: PLoS One. 2018 Apr 26;13(4):e0196595. doi: 10.1371/journal.pone.0196595 (PMC5919619; doi:10.1371/journal.pone.0196595)
Supplement: S7 Fig — (A) ROC curves of free fatty acids (FFAs) ω-hydroxyl palmitic acid (16OH-16:0), cerotic acid (26:0), and DHA (22:6); (B) Area under the curve (AUC) representation for the testing samples by partial least square—discriminant analysis (PLSA-DA) built with the three FFAs; (C) Predicted class probability for the testing set of samples of cpdm and WT epidermis. (DOCX) [file pone.0196595.s007.docx]

**
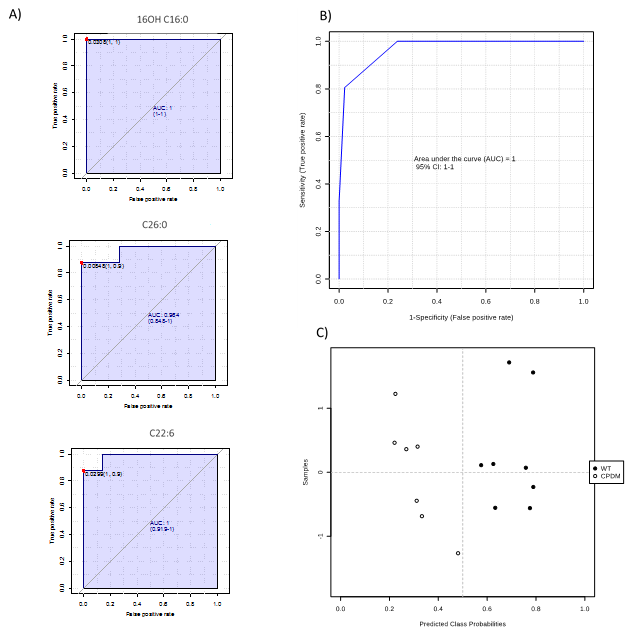
**

**S7 Fig. Discriminative value of a set of three free fatty acids.** (A) ROC curves of free fatty acids (FFAs) ω-hydroxyl palmitic acid (16OH-16:0), cerotic acid (26:0), and DHA (22:6); (B) Area under the curve (AUC) representation for the testing samples by partial least square – discriminant analysis (PLSA-DA) built with the three FFAs; (C) Predicted class probability for the testing set of samples of *cpdm* and WT epidermis.
