# Supplementary material for: Profiling of epidermal lipids in a mouse model of dermatitis: Identification of potential biomarkers
Source: PLoS One. 2018 Apr 26;13(4):e0196595. doi: 10.1371/journal.pone.0196595 (PMC5919619; doi:10.1371/journal.pone.0196595)
Supplement: S8 Fig — (A) ROC curves of sphingosine ceramides CerAS(d18:1/24:0)OH, CerAS(d18:1/16:0)OH and CerNS(d18:1/16:0) in nmol/mg of tissue. The threshold (red dotted line) set to differentiate between the two groups; (B) Area under the curve (AUC) representation for the testing samples by partial least square—discriminant analysis (PLSA-DA) built with the three selected ceramides; C) Predicted class probability for the testing set of samples of cpdm and WT epidermis. (DOCX) [file pone.0196595.s008.docx]

**A**


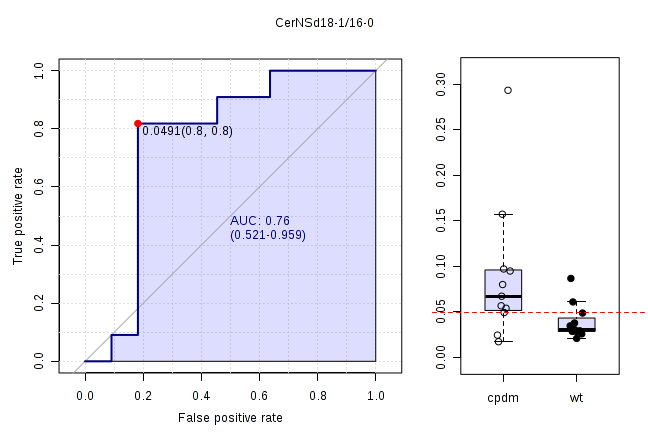

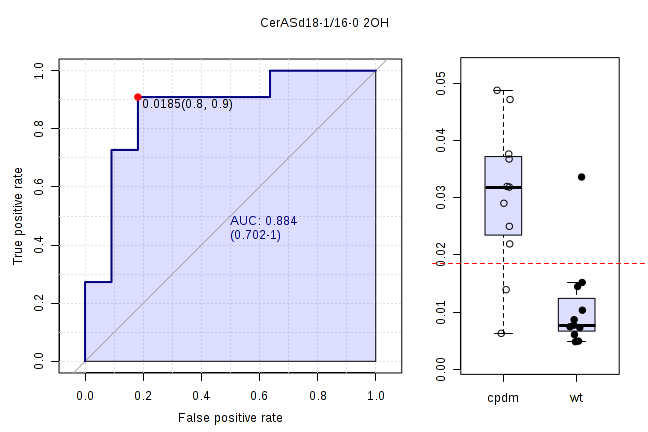


CerAS(d18:1/16:0)OH

CerAS(d18:1/16:0)


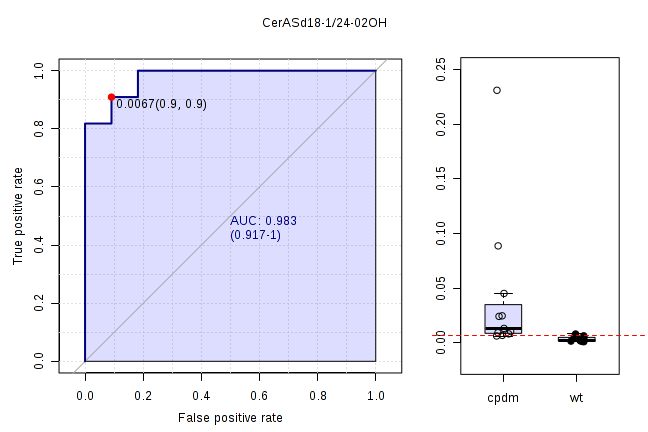


CerAS(d18:1/24:0)2 OH


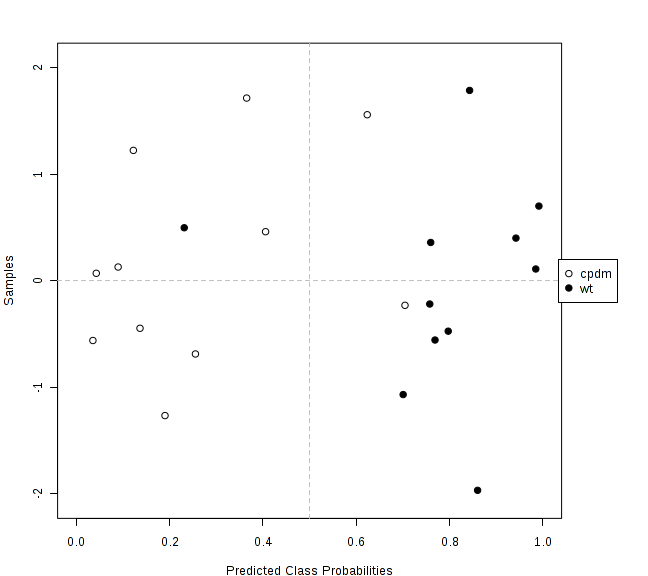

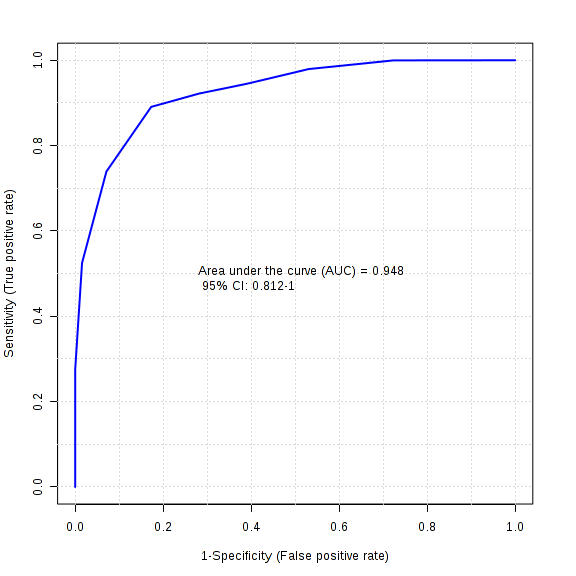


**A)**

**B)**

**C)**

**S8 Fig. Discriminative value of a set of three ceramides by LC-MS/MS.** (A) ROC curves of sphingosine ceramides CerAS(d18:1/24:0)OH, CerAS(d18:1/16:0)OH and CerNS(d18:1/16:0) in nmol/mg of tissue. The threshold (red dotted line) set to differentiate between the two groups; (B) Area under the curve (AUC) representation for the testing samples by partial least square – discriminant analysis (PLSA-DA) built with the three selected ceramides; C) Predicted class probability for the testing set of samples of *cpdm* and WT epidermis.
