# Supplementary material for: Profiling of epidermal lipids in a mouse model of dermatitis: Identification of potential biomarkers
Source: PLoS One. 2018 Apr 26;13(4):e0196595. doi: 10.1371/journal.pone.0196595 (PMC5919619; doi:10.1371/journal.pone.0196595)
Supplement: S1 Table — Multidimensional scan modes used for exploratory detection of lipids in cpdm and WT epidermis of the testing set. (DOCX) [file pone.0196595.s009.docx]

**S1 Table. Discovery scans.** Multidimensional scan modes used for exploratory detection of lipids in *cpdm* and WT epidermis of the testing set.

| SCAN MODE | DESCRIPTION | ION MODE | ION DETECTED | TARGETED LIPIDS | REFERENCE | DATA COLLECTION TIME/SAMPLE |
| --- | --- | --- | --- | --- | --- | --- |
| Prec 184 | Precursor ion scan for m/z 184 | (+) | [M+H]+ | Phosphatidylcholine (PC), alkelnyl-acyl PC (ePC), sphingomyelin (SM) and LysoPC | *Brugger et al., 1997* | 2min |
| NL 141 | Neutral loss of 141 mass units | (+) | [M+H]+ | Phosphatidylethanolamine (PE) | *Brugger et al., 1997* | 2min |
| NL 277 | Neutral loss of 277 mass units | (+) | [M+NH4]+ | Phosphatidylinositol (PI) | *Taguchi et al., 2005* | 2min |
| NL 185 | Neutral loss of 185 mass units | (+) | [M+H]+ | Phosphatydylserine (PS) | *Brugger et al., 1997* | 2min |
| Prec 369.1 | Precursor ion scan for m/z 369.1 | (+) | [M+NH4]+ | Cholesteryl esters (CE) | *Liebisch et al., 2006* | 2min |
| Prec 264.3 | Precursor ion scan for m/z 264.3 | (+) | [M+H]+ | Ceramides (d18:1; sphingosines)/Cerebrosides | *Colsch et al, 2004; Merrill et al., 2005* | 2min |
| Prec 266.4 | Precursor ion scan for m/z 266.4 | (+) | [M+H]+ | Ceramides (d18:0; Dihydro-ceramides) | *Colsch et al, 2004; Merrill et al., 2005* | 2min |
| Prec 282.4 | Precursor ion scan for m/z 282.4 | (+) | [M+H]+ | Ceramides (t18:0; phyto-ceramides) | *Colsch et al, 2004; Merrill et al., 2005* | 2min |
| NL 299 | Neutral loss of 299 mass units | (+) | [M+H]+ | Oleate (acyl residue) | *Li et al., 2014* | 2min |
| NL 321 | Neutral loss of 321 mass units | (+) | [M+H]+ | Arachidonate (acyl residue) | *Li et al., 2014* | 2min |
| Prec 85 | Precursor ion scan for m/z 85 | (+) | [M+H]+ | Acylcarnitines | *Han & Gross, 2005* | 2min |
| Prec 303.1 | Precursor ion scan for m/z 303.1 | (-) | [M-H]+ | Arachidonate (acyl residue) | *Li et al., 2014* | 2min |
| Single ion monitoring (SIM) | m/z expected for FFA | (-) | [M-H]+ | Free Fatty Acid profiles |  | 2min |
| MRM |  | (+) | [M+H]+ | Cholesteryl esters Profiling | *Chowedhury et al, 2016* | 2min |
| N/A | Full mass scan | (+) | [M+H]+ | Glycerolipids | *Brugger et al., 1997* | 2min |
| N/A | Full mass scan | (-) | [M-H]^-^ | Free fatty acids and glycerolipids | *Brugger et al., 1997* | 2min |
