# Supplementary material for: Profiling of epidermal lipids in a mouse model of dermatitis: Identification of potential biomarkers
Source: PLoS One. 2018 Apr 26;13(4):e0196595. doi: 10.1371/journal.pone.0196595 (PMC5919619; doi:10.1371/journal.pone.0196595)
Supplement: S2 Table — List of transitions in the MRM profiling method in positive ion mode (method 1) used to detect the relative amounts of lipids in the samples by the exploratory experiments. Each transition is represented by the m/z value of the parent ion, followed by the m/z value of the fragment released after collision at Q2. (DOCX) [file pone.0196595.s010.docx]

**S2 Table. MRM-profiling method in positive ion mode**. List of transitions in the MRM profiling method in positive ion mode (method 1) used to detect the relative amounts of lipids in the samples by the exploratory experiments. Each transition is represented by the *m/z* value of the parent ion, followed by the *m/z* value of the fragment released after collision at Q2.

| Lipid Class | Parent | Fragment | Lipid Class | Parent | Fragment | Lipid Class | Parent | Fragment |
| --- | --- | --- | --- | --- | --- | --- | --- | --- |
| Phosphatidylcholine (PC), alkelnyl-acyl PC (ePC), sphingomyelin (SM) and LysoPC | 496.2 | 184.1 | Phosphatidylinositol (PI) | 852.5 | 575.5 | Sphingo-sines | 694.4 | 264.3 |
|  | 520.2 | 184.1 |  | 854.5 | 577.5 |  | 873.7 | 264.3 |
|  | 522.2 | 184.1 |  | 876.5 | 599.5 |  | 875.1 | 264.3 |
|  | 689.8 | 184.1 |  | 878.5 | 601.5 |  | 875.6 | 264.3 |
|  | 703.8 | 184.1 |  | 880.5 | 603.5 | Dihydro-ceramides | 438 | 266.4 |
|  | 704.8 | 184.1 |  | 881.5 | 604.5 |  | 567.7 | 266.4 |
|  | 705.8 | 184.1 |  | 882.5 | 605.5 |  | 568.6 | 266.4 |
|  | 706.8 | 184.1 |  | 883.5 | 606.5 |  | 877.7 | 266.4 |
|  | 719.8 | 184.1 |  | 904.5 | 627.5 |  | 900.8 | 266.4 |
|  | 720.8 | 184.1 |  | 905.5 | 628.5 |  | 901.5 | 266.4 |
|  | 732.8 | 184.1 |  | 906.5 | 629.5 | Phytoceramides | 282.1 | 282.4 |
|  | 734.8 | 184.1 | Phosphatydylserine (PS) | 760.5 | 575.5 |  | 300.1 | 282.4 |
|  | 735.8 | 184.1 |  | 762.5 | 577.5 |  | 538.2 | 282.4 |
|  | 746.8 | 184.1 |  | 788.2 | 603.2 |  | 554.2 | 282.4 |
|  | 758.8 | 184.1 |  | 789.5 | 604.5 |  | 564 | 282.4 |
|  | 759.9 | 184.1 |  | 790.5 | 605.5 |  | 566.1 | 282.4 |
|  | 760.8 | 184.1 |  | 791.5 | 606.5 |  | 650.3 | 282.4 |
|  | 761.9 | 184.1 |  | 812.5 | 627.5 | Acylcarnitines | 359.25 | 85.1 |
|  | 782.8 | 184.1 |  | 814.5 | 629.5 |  | 372.2 | 85.1 |
|  | 784.8 | 184.1 |  | 816.5 | 631.5 |  | 376.2 | 85.1 |
|  | 786.9 | 184.1 |  | 836.5 | 651.5 |  | 386.3 | 85.1 |
|  | 787.9 | 184.1 |  | 844.5 | 659.5 |  | 398.3 | 85.1 |
|  | 788.9 | 184.1 |  | 846.5 | 661.5 |  | 400.3 | 85.1 |
|  | 815.9 | 184.1 |  | 872.5 | 687.5 |  | 401.3 | 85.1 |
| Phosphatidylethanolamine (PE) | 702.5 | 561.5 |  | 874.5 | 689.5 |  | 402.2 | 85.1 |
|  | 716.5 | 575.5 |  | 875.5 | 690.5 |  | 414.3 | 85.1 |
|  | 717.2 | 576.2 | Sphingosines | 536.1 | 264.3 |  | 415.25 | 85.1 |
|  | 718.5 | 577.5 |  | 537.9 | 264.3 |  | 424.2 | 85.1 |
|  | 719.5 | 578.5 |  | 538.2 | 264.3 |  | 426.3 | 85.1 |
|  | 730.5 | 589.5 |  | 539.2 | 264.3 |  | 427.4 | 85.1 |
|  | 740.5 | 599.5 |  | 554.2 | 264.3 |  | 428.3 | 85.1 |
|  | 742.5 | 601.5 |  | 566.2 | 264.3 |  | 429.3 | 85.1 |
|  | 743.5 | 602.5 |  | 622.1 | 264.3 |  | 442.25 | 85.1 |
|  | 744.5 | 603.5 |  | 622.35 | 264.3 |  | 454.3 | 85.1 |
|  | 745.5 | 604.5 |  | 648.4 | 264.3 |  | 456.3 | 85.1 |
|  | 746.5 | 605.5 |  | 649.8 | 264.3 |  | 460.3 | 85.1 |
|  | 747.5 | 606.5 |  | 650.4 | 264.3 |  | 484 | 85.1 |
|  | 758.5 | 617.5 |  | 651.4 | 264.3 |  | 531.2 | 85.1 |
|  | 766.5 | 625.5 |  | 666.35 | 264.3 |  |  |  |
|  | 768.5 | 627.5 |  | 678.4 | 264.3 |  |  |  |
|  | 769.5 | 628.5 |  | 679.4 | 264.3 |  |  |  |
|  | 770.5 | 629.5 |  | 694.15 | 264.3 |  |  |  |

**S2 Table:** Continuation

| Lipid Class | Parent | Fragment | Lipid Class | Parent | Fragment |
| --- | --- | --- | --- | --- | --- |
| Cholesteryl esters (CE) | 587.2 | 369.1 | Arachidonate (acyl residue) | 559.2 | 303.1 |
|  | 613.2 | 369.1 |  | 583.3 | 303.1 |
|  | 615.2 | 369.1 |  | 585.2 | 303.1 |
|  | 641.3 | 369.1 |  | 587 | 303.1 |
|  | 643.3 | 369.1 |  | 587.4 | 303.1 |
|  | 665.3 | 369.1 |  | 607.35 | 303.1 |
|  | 667.3 | 369.1 |  | 661.2 | 303.1 |
|  | 669.3 | 369.1 |  | 671.3 | 303.1 |
|  | 671.3 | 369.1 |  | 699.3 | 303.1 |
|  | 689.4 | 369.1 |  | 745.5 | 303.1 |
|  | 691.4 | 369.1 |  | 840.3 | 303.1 |
|  | 693.4 | 369.1 |  | 898.3 | 303.1 |
|  | 695.4 | 369.1 |  | 898.75 | 303.1 |
|  | 697.4 | 369.1 |  | 899.6 | 303.1 |
|  | 699.4 | 369.1 |  | 939.15 | 303.1 |
|  | 717.4 | 369.1 |  | 939.5 | 303.1 |
|  | 719.4 | 369.1 |  | 940.3 | 303.1 |
|  | 721.4 | 369.1 |  | 940.65 | 303.1 |
|  | 723.4 | 369.1 |  | 952.75 | 303.1 |
|  | 725.4 | 369.1 |  | 953.7 | 303.1 |
|  | 727.4 | 369.1 |  | 954.9 | 303.1 |
|  | 755.4 | 369.1 |  | 966.7 | 303.1 |
|  | 781.4 | 369.1 |  | 967.8 | 303.1 |
|  | 783.4 | 369.1 |  | 968.8 | 303.1 |
|  | 795.4 | 369.1 |  | 980.95 | 303.1 |
|  | 797.4 | 369.1 |  | 981.8 | 303.1 |
|  | 809.4 | 369.1 |  | 982.6 | 303.1 |
|  | 811.4 | 369.1 | Oleate (acyl residue neutral loss) | 846.5 | 547.5 |
|  | 823.4 | 369.1 |  | 848.5 | 549.5 |
|  | 825.4 | 369.1 |  | 849.5 | 550.5 |
|  | 837.4 | 369.1 |  | 850.5 | 551.5 |
|  | 839.4 | 369.1 |  | 851.5 | 552.5 |
| Arachidonate (acyl residue neutral loss) | 634.3 | 313.3 |  | 872.5 | 573.5 |
|  | 662.3 | 341.3 |  | 873.5 | 574.5 |
|  | 870.3 | 549.3 |  | 874.5 | 575.5 |
|  | 872.3 | 551.3 |  | 875.5 | 576.5 |
|  | 896.3 | 575.3 |  | 876.5 | 577.5 |
|  | 898.4 | 577.4 |  | 877.5 | 578.5 |
|  | 899.3 | 578.3 |  | 878.5 | 579.5 |
|  | 920.4 | 599.4 |  | 896.5 | 597.5 |
|  | 923.5 | 602.5 |  | 898.5 | 599.5 |
|  | 924.5 | 603.5 |  | 899.5 | 600.5 |
|  | 922.5 | 601.4 |  | 900.5 | 601.5 |
|  | 925.5 | 604.4 |  | 901.5 | 602.5 |
|  | 926.5 | 605.4 |  | 902.6 | 603.6 |
|  | 921.5 | 600.35 |  | 903.6 | 604.6 |
|  | 897.5 | 576.3 |  | 904.6 | 605.6 |
|  | 894.5 | 573.2 |  |  |  |
