# Supplementary material for: Profiling of epidermal lipids in a mouse model of dermatitis: Identification of potential biomarkers
Source: PLoS One. 2018 Apr 26;13(4):e0196595. doi: 10.1371/journal.pone.0196595 (PMC5919619; doi:10.1371/journal.pone.0196595)
Supplement: S3 Table — List of single ions monitored in the MRM profiling method in negative ion mode (method 2) used to detect the relative amounts of free fatty acids in the lipid extracts from samples. (DOCX) [file pone.0196595.s011.docx]

**S3 Table. MRM-profiling method in negative ion mode.** List of single ions monitored in the MRM profiling method in negative ion mode (method 2) used to detect the relative amounts of free fatty acids in the lipid extracts from samples.

| MRM | MRM | MRM |
| --- | --- | --- |
| 2155.2 -> 2155.2 | 395.4 -> 395.4 | 271.3 -> 271.3 |
| 2127.1 -> 2127.1 | 395.3 -> 395.3 | 256.3 -> 256.3 |
| 1864 -> 1864 | 393.3 -> 393.3 | 255.3 -> 255.3 |
| 1836.1 -> 1836.1 | 368.4 -> 368.4 | 253.3 -> 253.3 |
| 1601 -> 1601 | 367.4 -> 367.4 | 227.3 -> 227.3 |
| 1572.9 -> 1572.9 | 367.3 -> 367.3 | 225.2 -> 225.2 |
| 1544.9 -> 1544.9 | 365.3 -> 365.3 | 199.2 -> 199.2 |
| 1024.6 -> 1024.6 | 355.3 -> 355.3 | 197.2 -> 197.2 |
| 906.7 -> 906.7 | 353.3 -> 353.3 | 2181.3 -> 2181.3 |
| 904.7 -> 904.7 | 339.4 -> 339.4 |  |
| 902.7 -> 902.7 | 339.3 -> 339.3 |  |
| 890.7 -> 890.7 | 337.3 -> 337.3 |  |
| 888.7 -> 888.7 | 331.3 -> 331.3 |  |
| 886.6 -> 886.6 | 329.3 -> 329.3 |  |
| 878.6 -> 878.6 | 329.3 -> 329.3 |  |
| 876.6 -> 876.6 | 327.3 -> 327.3 |  |
| 874.6 -> 874.6 | 327.3 -> 327.3 |  |
| 864.6 -> 864.6 | 311.3 -> 311.3 |  |
| 862.6 -> 862.6 | 309.3 -> 309.3 |  |
| 860.6 -> 860.6 | 309.3 -> 309.3 |  |
| 852.6 -> 852.6 | 305.3 -> 305.3 |  |
| 850.6 -> 850.6 | 304.3 -> 304.3 |  |
| 834.6 -> 834.6 | 303.3 -> 303.3 |  |
| 832.6 -> 832.6 | 303.3 -> 303.3 |  |
| 822.6 -> 822.6 | 301.3 -> 301.3 |  |
| 806.6 -> 806.6 | 295.3 -> 295.3 |  |
| 804.5 -> 804.5 | 284.3 -> 284.3 |  |
| 794.7 -> 794.7 | 283.3 -> 283.3 |  |
| 778.5 -> 778.5 | 283.3 -> 283.3 |  |
| 507.5 -> 507.5 | 282.3 -> 282.3 |  |
| 487.5 -> 487.5 | 281.3 -> 281.3 |  |
| 479.4 -> 479.4 | 280.3 -> 280.3 |  |
| 465.4 -> 465.4 | 279.3 -> 279.3 |  |
| 451.4 -> 451.4 | 279.3 -> 279.3 |  |
| 423.4 -> 423.4 | 277.3 -> 277.3 |  |
| 417.4 -> 417.4 | 277.3 -> 277.3 |  |
| 396.4 -> 396.4 | 275.3 -> 275.3 |  |
