# Supplementary material for: Profiling of epidermal lipids in a mouse model of dermatitis: Identification of potential biomarkers
Source: PLoS One. 2018 Apr 26;13(4):e0196595. doi: 10.1371/journal.pone.0196595 (PMC5919619; doi:10.1371/journal.pone.0196595)
Supplement: S4 Table — Tentative attribution of lipids (confirmed by MS/MS experiments), area under the curve (AUC), p-values and log2 fold change (FC) for all ion pairs with AUC scores above 0.5. (DOCX) [file pone.0196595.s012.docx]

**S4 Table. Biomarker univariate analysis by ROC curve.** Tentative attribution of lipids (confirmed by MS/MS experiments), area under the curve (AUC), p-values and log2 fold change (FC) for all ion pairs with AUC scores above 0.5.

| Lipid tentative attribution or *m/z* | AUC | t-tests | Log2 FC |
| --- | --- | --- | --- |
| CerDS(Sphinganine) | 1 | 3.E-04 | 0.58 |
| CerNS(d18:1/16:1) | 1 | 2.E-06 | 0.57 |
| CerNS(d18:1/16:0) | 1 | 8.E-06 | 2.10 |
| CerAS(d18:1/160)2OH | 1 | 5.E-07 | 2.09 |
| CerAP(t18:0/16:0)2OH | 1 | 1.E-05 | 0.44 |
| CerNS(d18:1/22:0) | 1 | 2.E-07 | 0.59 |
| CerNS(d18:1/24:1) | 1 | 5.E-05 | 0.64 |
| CerAP(t18:0/22:0)2OH | 1 | 1.E-08 | 0.31 |
| CerNS(d18:1/24:0) | 1 | 7.E-08 | 1.65 |
| CerAS(d181/24:0)2OH | 1 | 1.E-08 | 1.08 |
| CerAS(d18:1/26:0)2OH | 1 | 1.E-07 | 0.60 |
| PG(32:2) / SM(d18:0/17:0) | 1 | 3.E-04 | 1.05 |
| SM(d18:0/20:00) / SM(d16:0/22:0) /possible isotope of PC(34:1) | 1 | 1.E-03 | 0.96 |
| PC(32:0) | 0.98214 | 7.E-03 | 0.97 |
| SM(d18:1/16:0) / SM(d16:1/18:0) | 0.96429 | 5.E-03 | 1.20 |
| ePC(34:1) / pPC(34:0) | 0.94643 | 1.E-03 | 0.34 |
| PC(34:1) | 0.94643 | 2.E-03 | 0.90 |
| PC(36:1) | 0.94643 | 6.E-04 | 0.54 |
| SM(24:0) | 0.94643 | 2.E-03 | 0.51 |
| DSM(16:0) | 0.92857 | 4.E-03 | 0.66 |
| SM(d16:1/24:0) / SM(d18:1/22:0) | 0.92857 | 6.E-04 | 0.96 |
| CerNP(t18:0/16:0) | 0.91071 | 2.E-03 | 0.44 |
| 662.3 NL AA | 0.91071 | 3.E-03 | 0.28 |
| PC(36:2) | 0.875 | 5.E-03 | 0.81 |
| C16:0 Carnitine | 0.85714 | 8.E-03 | 0.39 |
| C18:0 Carnitine | 0.83929 | 9.E-03 | 0.05 |
| ePC(32:0) | 0.83929 | 1.E-02 | 0.39 |
| PC(32:1) | 0.83929 | 1.E-02 | 0.29 |
| C10:1-OH Carnitine | 0.82143 | 7.E-02 | -0.42 |
| PC(34:2) | 0.82143 | 2.E-02 | 0.70 |
| C10:0 Carnitine | 0.80357 | 6.E-02 | -0.59 |
| C12:1-OH Carnitine | 0.80357 | 7.E-02 | -0.76 |
| CerNS(d18:1/18:0) | 0.80357 | 3.E-02 | 0.36 |
| 634.3 NL AA | 0.80357 | 2.E-02 | 0.06 |
| C12:0 Carnitine | 0.78571 | 7.E-02 | -1.01 |
| 402.2 Carnitine | 0.78571 | 8.E-02 | -0.30 |
| C14:0 Carnitine | 0.78571 | 9.E-02 | -0.83 |
| CerNS(d18:1/26:0) | 0.78571 | 3.E-02 | 0.25 |
| Sphingosine | 0.76786 | 4.E-02 | 0.14 |
| PS(36:2) | 0.76786 | 4.E-02 | 0.10 |
| LPC(18:1) | 0.75 | 6.E-02 | 0.55 |
| CerNP(t18:0/18:1) | 0.75 | 7.E-02 | -0.06 |
| 689.8 PC | 0.75 | 4.E-02 | -0.12 |
| PC363 | 0.75 | 1.E-01 | 0.20 |
| 898.3 AA | 0.75 | 8.E-02 | -0.01 |
| 922.5 NL AA | 0.75 | 1.E-01 | -0.01 |
| C14:1 Carnitine | 0.73214 | 1.E-01 | -1.08 |
| 282 CerP | 0.73214 | 1.E-01 | 0.02 |
| C12:1 Carnitine | 0.71429 | 1.E-01 | -0.46 |
| 531.2 carnitine | 0.71429 | 3.E-01 | -0.12 |
| 896.5 NL OA | 0.71429 | 2.E-01 | -0.01 |
| CerNP(t18:0/18:0) | 0.69643 | 2.E-01 | -0.10 |
| 899.5 NL OA | 0.69643 | 2.E-01 | -0.10 |
| C14:1-OH Carnitine | 0.67857 | 2.E-01 | -0.19 |
| LPC(16:0) | 0.67857 | 2.E-01 | 0.60 |
| CerEOS(23:0/d18:1/16:0) | 0.67857 | 3.E-01 | 0.00 |
| 898.5 NL OA | 0.67857 | 1.E-01 | -0.07 |
| C14:2 Carnitine | 0.66071 | 3.E-01 | -0.40 |
| 376.2Carnitine | 0.64286 | 8.E-01 | -0.01 |
| 900.5 NL OA | 0.64286 | 3.E-01 | -0.15 |
| CE (19:0) | 0.60714 | 4.E-01 | 0.01 |
| CerNDS(d18:0/18:0) | 0.58929 | 4.E-01 | -0.01 |
| PE (34:2) | 0.58929 | 5.E-01 | 0.00 |
| 873.5 NL OA | 0.58929 | 5.E-01 | -0.06 |
| 901.5 NL OA | 0.58929 | 4.E-01 | -0.10 |
| PE(36:3) | 0.57143 | 4.E-01 | -0.01 |
| PE(36:2) | 0.57143 | 8.E-01 | 0.00 |
| 872.5 NL OA | 0.53571 | 9.E-01 | 0.00 |
| 428.3 CE | 0.5 | 9.E-01 | 0.00 |
| 16OH-16:0 | 1 | 2.E-05 | 0.86 |
| 22:6 | 0.98214 | 3.E-04 | 0.53 |
| 20:4 | 0.96429 | 7.E-04 | 1.09 |
| 26:0 | 0.96429 | 2.E-02 | -1.11 |
| 874.6 | 0.96429 | 7.E-03 | -0.18 |
| 18:3 | 0.92857 | 2.E-03 | -0.29 |
| 487.5 | 0.92857 | 5.E-03 | -0.16 |
| 850.6 | 0.92857 | 6.E-03 | -0.28 |
| 876.6 | 0.92857 | 5.E-03 | -0.26 |
| 26.1 | 0.91071 | 3.E-03 | -0.19 |
| 32.0 | 0.89286 | 2.E-02 | -0.20 |
| 22:4 | 0.875 | 9.E-03 | 0.15 |
| 24:1 | 0.85714 | 2.E-02 | -0.16 |
| 878.6 | 0.85714 | 1.E-02 | -0.27 |
| 14:0 | 0.83929 | 3.E-02 | -0.11 |
| 18:4 | 0.82143 | 3.E-02 | -0.12 |
| 822.6 | 0.82143 | 3.E-02 | -0.23 |
| 890.7 | 0.82143 | 2.E-02 | -0.13 |
| 10:0 | 0.80357 | 6.E-02 | -0.10 |
| 20:0 | 0.80357 | 9.E-02 | -0.68 |
| 888:7 | 0.80357 | 5.E-02 | -0.11 |
| 417.4 | 0.78571 | 6.E-02 | -0.08 |
| 860.6 | 0.78571 | 5.E-02 | -0.14 |
| 902.7 | 0.78571 | 3.E-02 | -0.10 |
| 906.7 | 0.78571 | 4.E-02 | -0.12 |
| 852.6 | 0.76786 | 8.E-02 | -0.12 |
| 862.6 | 0.76786 | 6.E-02 | -0.15 |
| 886.6 | 0.76786 | 7.E-02 | -0.11 |
| 16;1 | 0.75 | 2.E-01 | -0.13 |
| 904.7 | 0.75 | 9.E-02 | -0.09 |
| 28:0 | 0.73214 | 1.E-01 | -0.13 |
| 20:3 | 0.73214 | 2.E-01 | 0.09 |
| ChS | 0.71429 | 2.E-01 | -0.38 |
| 23:0 | 0.67857 | 3.E-01 | 0.11 |
| 16:0 | 0.66071 | 2.E-01 | -0.20 |
| 34:0 | 0.66071 | 2.E-01 | -0.09 |
| 778.5 | 0.66071 | 5.E-01 | -0.17 |
| 18:0 | 0.64286 | 4.E-01 | -0.23 |
| 22:5 | 0.64286 | 4.E-01 | -0.33 |
| 834.6 | 0.64286 | 2.E-01 | -0.06 |
| 18:1 | 0.625 | 4.E-01 | -0.12 |
| 30:0 | 0.625 | 2.E-01 | -0.08 |
| 864.6 | 0.625 | 2.E-01 | -0.07 |
| 24:0 | 0.60714 | 3.E-01 | -0.21 |
| 22:1 | 0.57143 | 4.E-01 | -0.05 |
| 22:0 | 0.51786 | 7.E-01 | 0.03 |
| 24:6 | 0.51786 | 9.E-01 | 0.02 |
| 832.6 | 0.5 | 5.E-01 | -0.05 |
