# Supplementary material for: Profiling of epidermal lipids in a mouse model of dermatitis: Identification of potential biomarkers
Source: PLoS One. 2018 Apr 26;13(4):e0196595. doi: 10.1371/journal.pone.0196595 (PMC5919619; doi:10.1371/journal.pone.0196595)
Supplement: S5 Table — Class prediction of the validation set of samples by ROC based on potential ceramide biomarkers. (DOCX) [file pone.0196595.s013.docx]

**S5 Table. Class prediction by ROC curve selected ceramides.** Class prediction of the validation set of samples by ROC based on potential ceramide biomarkers.

| New sample name | Real class | Random Forrest | | PLS-DA | |
| --- | --- | --- | --- | --- | --- |
|  |  | Probability | Predicted class | Probability | Predicted class |
| CPDM8 | CPDM | 1 | CPDM | 0.90 | CPDM |
| CPDM9 | CPDM | 1 | CPDM | 0.66 | CPDM |
| CPDM10 | CPDM | 1 | CPDM | 0.79 | CPDM |
| CPDM11 | CPDM | 1 | CPDM | 0.89 | CPDM |
| CPDM12 | CPDM | 1 | CPDM | 0.83 | CPDM |
| CPDM13 | CPDM | 1 | CPDM | 0.80 | CPDM |
| CPDM14 | CPDM | 1 | CPDM | 0.78 | CPDM |
| CPDM15 | CPDM | 1 | CPDM | 0.71 | CPDM |
| CPDM16 | CPDM | 1 | CPDM | 0.89 | CPDM |
| CPDM17 | CPDM | 1 | CPDM | 0.85 | CPDM |
| WT9 | WT | 1 | WT | 0.65 | WT |
| WT10 | WT | 1 | WT | 0.67 | WT |
| WT11 | WT | 1 | WT | 0.64 | WT |
| WT12 | WT | 1 | WT | 0.64 | WT |
| WT13 | WT | 1 | WT | 0.68 | WT |
| WT14 | WT | 1 | WT | 0.67 | WT |
| WT15 | WT | 1 | WT | 0.66 | WT |
| WT16 | WT | 1 | WT | 0.68 | WT |
| WT17 | WT | 1 | WT | 0.71 | WT |
| WT18 | WT | 1 | WT | 0.66 | WT |
| WT19 | WT | 1 | WT | 0.69 | WT |
